# Restoration of ancestral transcriptional plasticity contributes to plastic heterosis in fatty liver of hybrid ducks

**DOI:** 10.1038/s42003-026-10049-7

**Published:** 2026-04-14

**Authors:** Ming-Min Xu, Yinhua Huang, Ya-Ping Zhang, Min-Sheng Peng

**Affiliations:** 1 Center for RNA Medicine, the Fourth Affiliated Hospital of School of Medicine, and International School of Medicine, International Institutes of Medicine, Zhejiang University, Yiwu, China; 2 State Key Laboratory of Genetic Evolution & Animal Models and Yunnan Key Laboratory of Molecular Biology of Domestic Animals, Kunming Institute of Zoology, Chinese Academy of Sciences, Kunming, China; 3 Kunming College of Life Science, University of Chinese Academy of Sciences, Kunming, China; 4 State Key Laboratory for Farm Animal Biotech Breeding, College of Biology Sciences, China Agricultural University, Beijing, China; 5 Bio-X Center for Interdisciplinary Innovation, Yunnan University, Kunming, China

**Keywords:** Evolutionary genetics, Gene expression

## Abstract

Phenotypic plasticity enables organisms to modify their phenotypes in response to environmental fluctuations. Interspecific hybrids can inherit parental phenotypic plasticity and even exhibit enhanced physiological performance under analogous environmental shifts, a phenomenon termed as plastic heterosis. However, the genetic mechanisms underlying plastic heterosis remain poorly understood. Here, we explore the transcriptional profiling of overfeeding-induced fatty liver in Peking ducks (*Anas platyrhynchos*), Muscovy ducks (*Cairina moschata*), and their reciprocal hybrids. We find that genes associated with fatty acid catabolism retained their ancestral plasticity in Muscovy ducks but lost it in Peking ducks. The parental transcriptional plasticity is reshuffled in hybrid ducks during hybridization. Specifically, the expression pattern of 129 Peking-derived alleles is converted to those of Muscovy-derived alleles in both reciprocal hybrid ducks in response to overfeeding. Among these, eight fatty acid catabolism-related alleles that had lost plasticity in Peking ducks restore ancestral plasticity in hybrids. Our findings systematically characterize transcriptional changes linked to parental phenotypic plasticity and illuminate its evolutionary trajectory. In particular, the restoration of ancestral plasticity in interspecific hybrids may represent a mechanism contributing to plastic heterosis.

## Abstract (web summary)

Evolutionary transcriptomics reveals mechanisms underlying plastic heterosis in hybrid ducks during fatty liver formation

## Introduction

Heterosis, frequently observed in interspecific hybrids, is characterized by traits that are superior to those of either parental species^1–3^. Classical genetic models attribute heterosis to interactions between paternal and maternal alleles in hybrids––either at the same loci via dominance or overdominance, or across different loci through epistasis^2,4,5^. Quantitative and population genetic studies have identified numerous associated quantitative trait loci (QTLs) and proposed dominance as a primary contributor to heterosis^6,7^. Concurrently, transcriptomic analyses have uncovered widespread non-additive gene expression in hybrids, wherein expression levels deviate from the mid-parental values, contributing to heterotic phenotypes^8^. For instance, gene expression dominance from either parent in hybrids has been implicated in the manifestation of heterosis^9^. Such non-additive expression patterns reflect extensive remodeling of parental gene patterns in hybrids^10,11^, most likely resulting from novel *cis*- and *trans*-regulatory interactions that emerge in hybrids following merging of divergent parental genomes during hybridization^12–14^. Beyond global gene expression changes, emerging studies indicated that allele-specific expression, the imbalanced expression between parental alleles, in hybrids also plays a crucial role in heterosis^15–17^. Together, these advances underscore the importance of transcriptional patterns in hybrids for understanding the molecular mechanisms underlying heterosis.

Plastic heterosis has been interpreted as environment-dependent heterosis in the context of phenotypic plasticity^18^, which describes an organism’s ability to generate variable phenotypes in response to environmental changes^19–22^. During interspecific hybridization, parental phenotypic plasticity itself can be reshaped in the offspring^18,23,24^. Consequently, interspecific hybrids tend to exhibit enhanced plasticity when the parental species display strong environmentally responsive traits, a phenomenon defined as plastic heterosis^23^. Gene expression is highly responsive to environmental variation, and genotype-by-environment (G×E) interactions account for a substantial proportion of gene expression variability^25^. The capacity of genes to alter their expression levels in response to environmental changes is referred to as transcriptional (or gene expression) plasticity^26,27^. As plastic heterosis represents a combination of heterosis and phenotypic plasticity, it is reasonable to hypothesize that the transcriptional plasticity of parental alleles may significantly influence heterotic phenotypes. However, few studies have directly linked gene- or allele-level expression plasticity to environment-induced heterotic phenotypes. It remains an open question how the parental transcriptional plasticity is reshaped in hybrids and how this, in turn, influences plastic heterosis in the progeny.

The waterfowl, such as ducks and geese, have evolved to develop non-pathogenic fatty livers spontaneously for energy storage by overconsumption in preparation for migration in the wild^28^. Dietary manipulation can simulate this phenotypic plasticity through overfeeding experiments (i.e., environmental changes) in Peking ducks (*Anas platyrhynchos*) and Muscovy ducks (*Cairina moschata*), which diverged from their last common ancestor (LCA) approximately 13.8 million years ago^29,30^. Muscovy ducks develop heavier fatty livers than Peking ducks following overfeeding^31,32^. Interestingly, their reciprocal interspecific hybrids, i.e., Mule ducks (the sterile hybrids from female Peking × male Muscovy) and Hinny ducks (the sterile hybrids from female Muscovy × male Peking), exhibited heterosis in developing fatty liver in response to overfeeding (Supplementary Fig. 1)^31^. And the Mule ducks have been widely used in the *foie gras* production^33^. The overfeeding-induced fatty liver in these two duck species and their interspecific hybrids provides a model of dietary manipulation to address potential roles of parental plasticity involved in plastic heterosis.

**Fig. 1:**
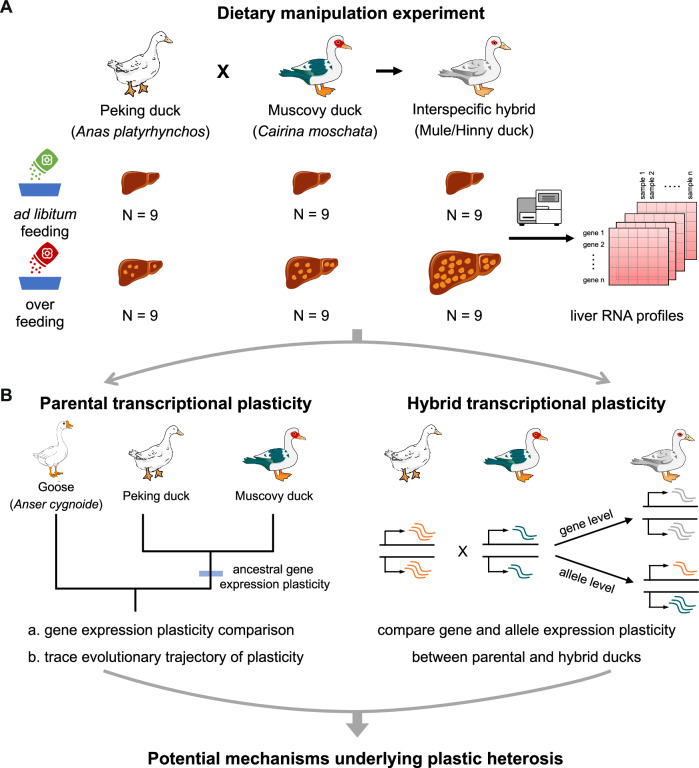
Overview of comparative transcriptomic analyses in parental and hybrid ducks to investigate the evolution of parental gene expression plasticity and its potential role in plastic heterosis during fatty liver development. **A** Muscovy ducks exhibit a heavier fatty liver than Peking ducks in response to overfeeding. Their interspecific reciprocal hybrids, Mule and Hinny ducks, display plastic heterosis, developing increased fatty liver under the same dietary stimuli. This trio-bins system constitutes a dietary manipulation experiment to investigate phenotypic plasticity and plastic heterosis. We reanalyzed previously published transcriptomic data from these three genotype ducks under *ad libitum* and overfed conditions, with each group consisting of nine biological replicates. **B** Using inferred ancestral expression plasticity, we compared differences in gene expression plasticity between parental ducks to depict evolutionary scenarios of phenotypic plasticity associated with varying degrees of fatty liver development. In hybrids, we comprehensively investigated plasticity by comparing gene- and allele-level expression plasticity with that of parental ducks.

Herein, we comprehensively reanalyzed the hepatic transcriptomic dataset from a previous study that examined these four genotypes (Peking, Muscovy, Mule, and Hinny ducks) under both fed *ad libitum* and overfed conditions (i.e., simulating environmental changes) but did not explicitly address phenotypic plasticity and plastic heterosis^32^. We conducted a comparative transcriptomic analysis to characterize hepatic transcriptomic plasticity in parental duck species and their hybrids (Fig. 1). Our results provide insights into the evolutionary dynamics of phenotypic plasticity and the mechanisms driving plastic heterosis.

## Results

### Divergence in gene expression plasticity between parental ducks

The differing susceptibility to fatty liver between Peking and Muscovy ducks indicates that the phenotypic plasticity underlying fatty liver development is evolvable. To explore the relationship between gene expression plasticity and evolved gene expression differences, we examined the correlation between the expression plasticity of Peking ducks and the total expression divergence between Peking and Muscovy ducks (Materials and methods). We found that the correlation between expression plasticity and evolutionary divergence was substantially more positive than correlations generated from a randomization test (Supplementary Fig. 2).

Genes whose plastic responses occurred in the same direction as evolutionary divergence were classified as likely adaptive, whereas those showing plasticity in the opposite direction were classified as nonadaptive^34^.We identified a substantially larger number of genes exhibiting adaptive plasticity (*n* = 2753) compared to those displaying nonadaptive plasticity (*n* = 874), suggesting that gene expression plasticity parallels evolutionary divergence of gene expression between species and may be adaptive.

**Fig. 2:**
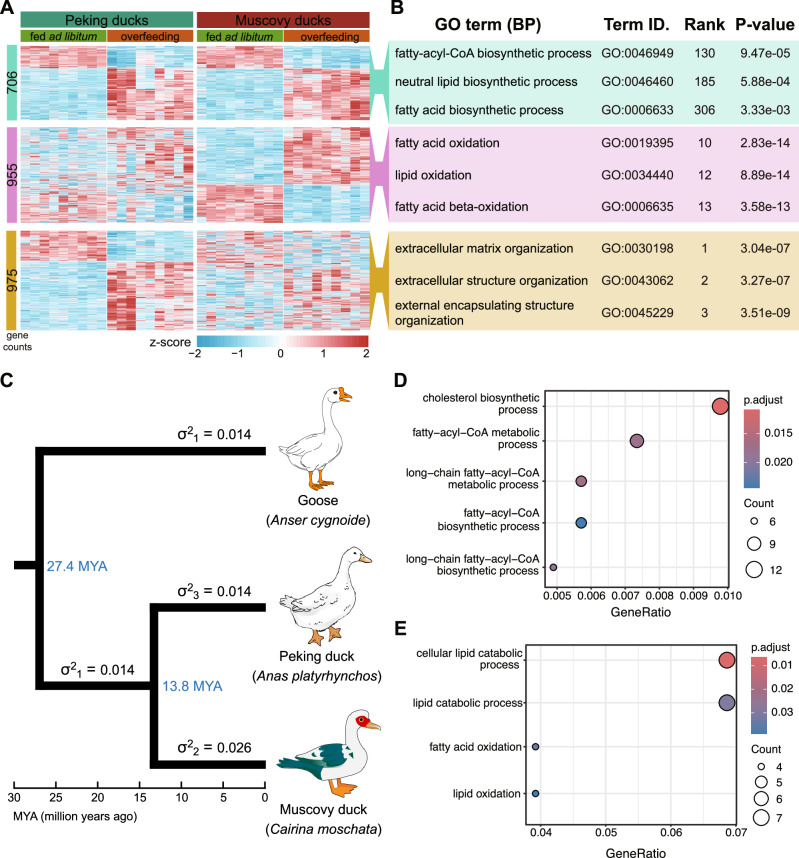
Transcriptional plasticity profiles in parental duck species. **A** Heatmaps showing the expression of plastic genes shared between Peking and Muscovy ducks (top), specific to Muscovy ducks (middle), and specific to Peking ducks (bottom). Expression values were transformed using the variance-stabilizing transformation and scaled per gene by z-score to highlight relative expression differences across samples. The color bar indicates scaled expression, with red representing relatively higher expression and blue representing relatively lower expression. **B** Selected enriched Gene Ontology (GO) terms associated with shared plastic genes between Peking and Muscovy ducks (top), downregulated plastic genes specific to Muscovy ducks (middle), and plastic genes specific to Peking ducks (bottom). **C** Evolutionary rates of hepatic transcriptome for Peking and Muscovy ducks. The evolutionary rate parameter (*σ*_2_) was estimated and labeled on each branch leading to Peking or Muscovy duck lineages, with geese liver transcriptome treated as an outgroup. Divergence times among species were obtained from the TimeTree database (http://www.timetree.org/) and are indicated in blue. MYA million years ago. Dot plots displaying enriched GO terms for ancestral plastic genes that were upregulated (**D**) or downregulated (**E**).

To further account for the varying degrees of parental phenotypic plasticity in fatty liver development, we characterized transcriptional plasticity in Peking and Muscovy ducks by identifying plastic genes—those exhibiting significant differences in expression between *ad libitum* feeding and overfeeding conditions. In total, 706 plastic genes were shared between Peking and Muscovy ducks (Fig. 2A). Functional enrichment analysis revealed that these plastic genes were significantly associated with biosynthetic pathways of fatty acids (Fig. 2B; Supplementary Data 1). Several key lipogenic genes (e.g., *SCD*, *ACLY*, *ELOVL6*, *ELOVL7*, and *DGAT2*) were upregulated in response to overfeeding in both duck species. We identified 975 and 955 plastic genes exhibiting species-specific transcriptional plasticity in Peking and Muscovy ducks, respectively (Fig. 2A). For the 955 plastic genes specific to Muscovy ducks, only downregulated plastic genes were significantly enriched in lipid and fatty acid catabolic processes (Supplementary Data 2). We further found that the fatty acid and lipid oxidation pathway were downregulated in overfed Muscovy duck (Supplementary Fig. 3). These results are consistent with previous reports showing that fatty acid and lipid oxidation pathways were transcriptionally reduced in the livers of overfed Muscovy ducks^32,35^. In contrast, plastic genes specific to Peking ducks showed limited functional enrichment related to lipid metabolism and catabolism (Fig. 2B). These findings suggest that upregulation of fatty acid biosynthesis represents a conserved plastic response to overfeeding in both species, whereas a reduction in fatty acid oxidation appears to be a Muscovy duck-specific mechanism contributing to fatty liver formation.

To trace the evolutionary dynamic of gene expression plasticity between Peking and Muscovy ducks, the evolutionary relationship of the hepatic transcriptome was first inferred based on a well-established phylogeny using geese (*Anser anser* or *Anser cygnoides*) as an outgroup. We used CAGEE^36^ to model the rate of gene expression changes per unit evolutionary time (*σ*²) and then quantified the evolutionary divergence of hepatic transcriptomes. The hepatic transcriptome of Muscovy ducks evolved at a faster rate compared to both Peking ducks and geese, which exhibited similar evolutionary rates (Fig. 2C; *σ*^2^_Muscovy_: 0.026, *σ*^2^_Peking_: 0.014, and *σ*^2^_goose_: 0.014). Next, we reconstruct the evolutionary trajectory of diverged transcriptional plasticity associated with fatty acid catabolism through inference of ancestral transcriptional plasticity in the LCA of Peking and Muscovy ducks. The ancestral plastic genes were defined as those exhibiting at least a twofold difference in inferred expression between two feeding conditions. In total, 1,655 ancestral plastic genes were identified, including 1,513 upregulated and 142 downregulated genes. Interestingly, ancestral plastic genes that were upregulated were enriched in fatty acid biosynthesis pathways, whereas those that were downregulated were associated with fatty acid oxidation (Fig. 2D, E; Supplementary Fig. 4 and Supplementary Data 3). These results suggest that the upregulation of fatty acid synthesis and the downregulation of fatty acid oxidation were likely ancestral responses to excess energy intake. This transcriptional strategy was largely retained in Muscovy ducks but was almost completely lost in Peking ducks following their divergence from the LCA.

### The profiling of gene expression plasticity in hybrid ducks

Given the divergence in plasticity responsive to overfeeding between parental duck species, we sought to understand how parental gene expression plasticity is remodeled in hybrids during interspecific hybridization. Our analysis revealed that the gene expression profiles of hybrid ducks were more strongly correlated with those of Peking ducks than with those of Muscovy ducks (Supplementary Fig. 5). Consistent with this, we found that the number of differentially expressed genes (DEGs) between Muscovy ducks and hybrid ducks was greater than that between Peking ducks and hybrid ducks. However, only a small subset of genes showed significantly differential expression between the parental ducks and hybrids under both *ad libitum* and overfed conditions (Supplementary Table 1). These findings suggest that the extent of parental gene expression remodeling in hybrids is moderate, with parental gene expression patterns exerting a preferential influence on shaping the transcriptional profiles of hybrids.

**Fig. 3:**
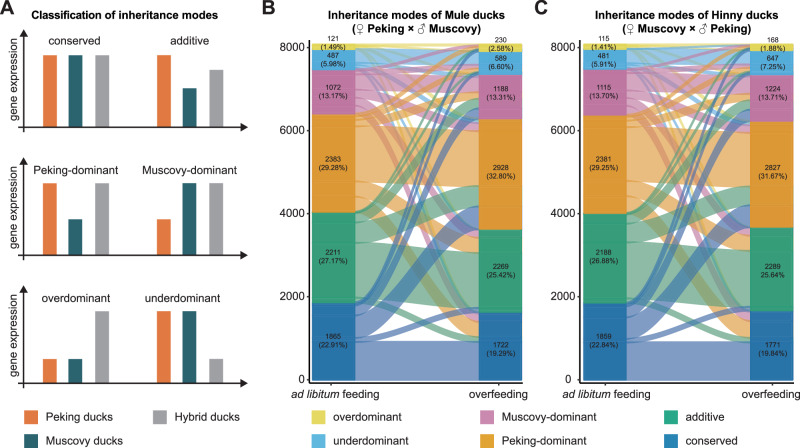
Transcriptional plasticity profiles in interspecific hybrid ducks. **A** Schematic illustrating hypothetical patterns of gene expression in Peking, Muscovy, and hybrid ducks, depicting conserved, additive, dominant, underdominant, and overdominant modes of inheritance. Alluvial plots showing changes in the number of genes assigned to each inheritance mode of gene expression across the two feeding conditions for Mule (**B**) and Hinny (**C**) ducks. The number of genes in each inheritance mode is shown in black, with their proportions indicated as black percentages in brackets.

To further characterize gene expression differences between parental and hybrid ducks, we classified the modes of inheritance for gene expression in hybrid ducks into three categories: conserved, additive, and non-additive (comprising parents-dominance, overdominance, and underdominance) (Fig. 3A). The ratio of genes exhibiting different inheritance modes showed minimal variation between two feeding conditions (Fig. 3B, C), indicating that environmental effects had limited impact on the global pattern of inheritance modes of gene expression in hybrids. When comparing the magnitude of gene expression changes for Peking- and Muscovy-dominant genes between *ad libitum* and overfed conditions, we found few significant differences in effect size (Supplementary Fig. 6). These results suggest that Peking- and Muscovy-dominant genes may play similar roles in gene expression plasticity in hybrid ducks. For genes that exhibited changes in inheritance modes, we found that Muscovy-dominant genes were more likely to shift inheritance modes between feeding conditions compared with Peking-dominant genes (Fig. 3B, C; Chi-square test; Mule ducks, *p* value < 2.2 × 10^−16^; Hinny ducks, *p* value < 2.2 × 10^−16^), indicating that Muscovy-dominant genes were more sensitive to feeding conditions. It raises the possibility that alleles from Peking- and Muscovy-dominant genes in hybrids may exhibit varying levels of expression plasticity in response to feeding conditions.

**Fig. 4:**
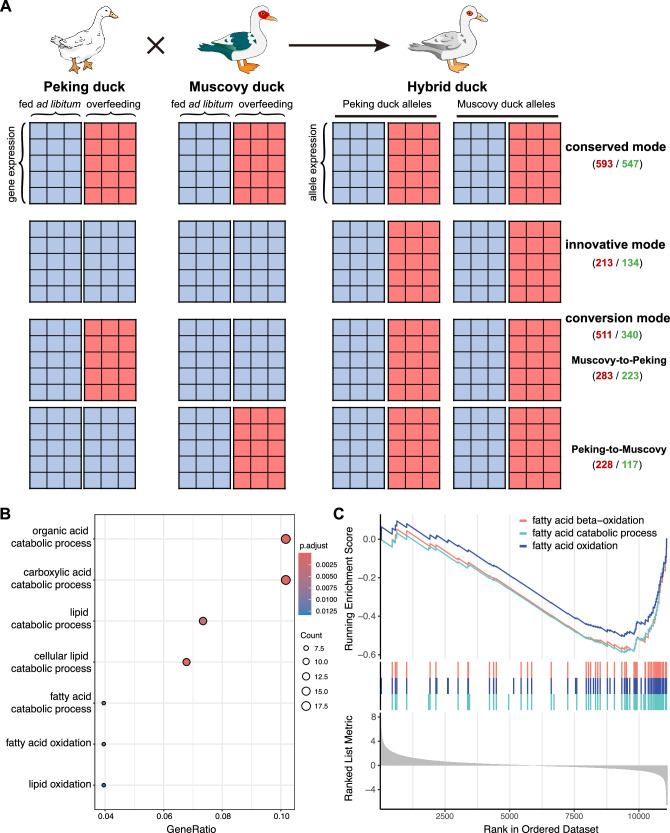
Transcriptional plasticity profiles in hybrid ducks. **A** Plastic Parental Alleles (PPAs) exhibit similar transcriptional plasticity in hybrid ducks, where both parental alleles were differentially expressed and shared the same regulation direction. The transcriptional plasticity patterns of PPAs were compared with their corresponding genes in parental species and categorized into three distinct modes: (1) conserved mode: PPAs display similar transcriptional plasticity to both parental genes (top rows). (2) innovative mode: PPAs exhibit transcriptional plasticity not observed in both parental genes (second rows). (3) conversion mode: only one gene of orthologous gene pairs exhibits plasticity in parental ducks, while the allele of the other non-plastic gene appears to be converted to the allele of the plastic gene, thereby gaining similar transcriptional plasticity in hybrid ducks. This mode was further specified as Muscovy-to-Peking (converted alleles from Muscovy ducks; third rows) and Peking-to-Muscovy (converted alleles from Peking ducks; bottom rows) mode. The numbers in brackets indicate the number of detected PPAs for each categorized mode in Mule ducks (red) and Hinny ducks (green). **B** Selected enriched GO terms relevant to lipid catabolic process for Peking-to-Muscovy alleles in Mule ducks. **C** Gene set enrichment analysis (GSEA) revealed that the lipid catabolism pathway was downregulated for Peking-to-Muscovy PPAs in Mule ducks.

In hybrid ducks, alleles showing significant expression differences between *ad libitum* and overfed conditions were defined as plastic alleles. Among these, alleles that exhibited consistent regulatory direction across feeding conditions were designated as Plastic Parental Alleles (PPAs). To investigate modes of parental allele plasticity, PPAs were classified into three distinct categories based on comparisons between their regulatory patterns in hybrids and those in the parental species (Fig. 4A). For example, in Mule ducks, we identified 1317 PPAs, compared to 706 genes exhibiting the same regulatory directions in both parental species. Among the PPAs, 593 maintained plasticity similar to that of the parental species, representing the conserved mode. In addition, 213 PPAs exhibited an innovative mode, characterized by the gain of plasticity absent in both parental species. The remaining 38.8% of PPAs (511 out of 1317) followed a conversion mode, in which a non-plastic allele from one parent was converted to the plastic allele from the other parent, resulting in similar plasticity in hybrid ducks. Specifically, 283 non-plastic alleles from Muscovy ducks were converted to their plastic Peking duck counterparts, forming M2P (Muscovy-to-Peking) PPAs. Conversely, 228 non-plastic alleles were shifted from Peking ducks to their plastic Muscovy duck counterparts, as P2M (Peking-to-Muscovy) PPAs (Fig. 4A). These distinct plasticity modes suggest that transcriptional plasticity of parental genes has been reshaped in hepatic transcriptomes of hybrid ducks during hybridization.

### Genetic mechanism underlying plastic heterosis

Our analysis on liver weight statistics revealed that hybrid ducks exhibit pronounced plastic heterosis in fatty liver formation (Supplementary Fig. 7; Supplementary Table 2). Although gene expression profiles of hybrid ducks more closely resembled those of Peking ducks than of Muscovy ducks (Supplementary Fig. 5), hybrid ducks displayed a propensity for fatty liver development comparable to that observed in Muscovy ducks. This apparent discordance between transcriptional similarity and phenotypic resemblance prompted us to investigate the genetic mechanisms underlying plastic heterosis in hybrid ducks. We focused on parent-dominance genes to evaluate whether canonical non-additive models of heterosis could explain plastic heterosis by associating gene expression plasticity with heterotic phenotypes. In total, we identified 369 Muscovy-dominant genes and 1325 Peking-dominant genes with consistent inheritance modes across feeding conditions in Mule ducks, and 419 Muscovy-dominant genes and 1295 Peking-dominant genes in Hinny ducks. However, functional enrichment analysis revealed that these gene sets showed limited enrichment for lipid metabolism-related pathways, hinting that non-additive effects were not the primary contributors to plastic heterosis in hybrid ducks.

We next sought to investigate whether transcriptional plasticity at the allele level might underlie plastic heterosis in hybrids. To explore the role of PPAs in plastic heterosis, we performed functional enrichment analyses for PPAs in Mule ducks. We found that conserved PPAs exhibited functional enrichment patterns similar to those of plastic genes shared between parental species, and that innovative PPAs were significantly enriched in biological processes related to development and morphogenesis. Among converted PPAs, P2M PPAs were significantly enriched in pathways associated with lipid and fatty acid catabolism (Fig. 4B). In contrast, M2P PPAs exhibited few enrichments in lipid and fatty acid catabolic pathways. Further gene set enrichment analysis (GSEA) revealed that biological pathways associated with fatty acid catabolism, such as GO:0009062 “fatty acid catabolic process,” GO:0034440 “lipid oxidation,” and GO:0006635 “fatty acid beta-oxidation”, were significantly downregulated for Peking-derived alleles within Mule ducks in response to overfeeding (Fig. 4C). All analysis results for the Hinny ducks were largely in agreement with those in the Mule ducks (Supplementary Figs. 8 and 9). Collectively, these findings suggest that the conversion of Peking-origin alleles to Muscovy-origin alleles, resulting in P2M PPAs, likely contributes to plastic heterosis in fatty liver formation in hybrid ducks.

To explore potential regulatory mechanisms underlying the conversion of plasticity in hybrids, we compared co-transcriptional modules identified by WGCNA (weighted gene co-expression network analysis)^37^ between parental and hybrid ducks. Among the eight modules identified in parental ducks, the brown module showed a strong negative association with overfed Muscovy ducks, whereas its association with overfed Peking ducks was much weaker and not statistically significant (Supplementary Fig. 10A). The brown-module genes were significantly enriched for lipid and fatty acid oxidation-related processes (Supplementary Fig. 10B), indicating that coordinated regulation of fatty acid oxidation genes is closely associated with fatty liver development in Muscovy ducks but largely absent or attenuated in Peking ducks. For hybrid ducks, WGCNA identified four co-expression modules. The blue module showed significant negative correlations with overfed Hinny and Mule ducks (Supplementary Fig. 10C). Within this module, 2210 pairs of Peking- and Muscovy-derived alleles were identified. Functional enrichment analysis revealed that genes corresponding to these allelic pairs were significantly enriched for lipid and fatty acid oxidation processes (Supplementary Fig. 10D), proposing Peking- and Muscovy-derived alleles relevant to lipid and fatty acid oxidation formed a shared co-expression module and were coordinately regulated in response to overfeeding in hybrids.

### Candidate functional genes for plastic heterosis of fatty liver

**Fig. 5:**
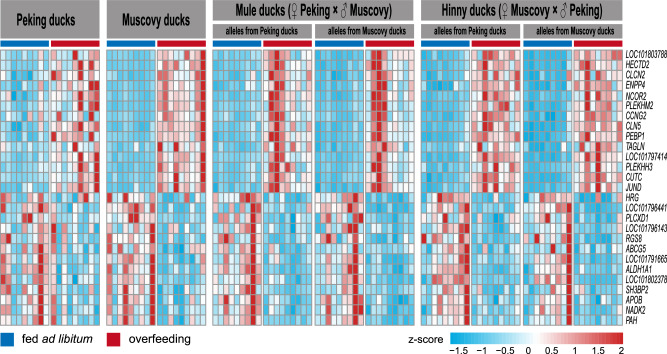
Expression of potential candidate genes involved in plastic heterosis. The heatmap illustrates the expression changes of Peking-to-Muscovy PPAs in both parental and hybrid ducks. A total of 27 genes exhibiting adaptive plasticity are displayed under two different feeding conditions. In Mule and Hinny ducks, the expression levels of these separate genes were visualized for each allele, distinguished by their species of origin.

We focused on P2M PPAs to identify candidate plastic genes for plastic heterosis. To alleviate potential effects induced by reciprocal crosses, we retained 129 genes after intersecting P2M PPAs from both Mule and Hinny ducks. GO enrichment analysis highlighted the significant role of these genes in lipid and fatty acid catabolism (Supplementary Data 4). Among the 129 genes, 27 genes demonstrated adaptive plasticity in parental species (Fig. 5), suggesting their positive contribution to the adaptation. Through a manual literature retrieval, we found four genes (i.e., *ABCG5*, *ALDH1A1*, *APOB*, and *NADK2*) were substantially relevant to lipid metabolism. Notably, both parental alleles of these genes were downregulated in the liver of hybrid ducks in response to overfeeding. The RNA-seq reads of these four genes were evenly distributed across exonic regions, indicating that their expression changes were unlikely due to localized mapping artifacts (Supplementary Fig. 11). Taken together, these genes involved in the process of lipid metabolism were inclined to be susceptible to overfeeding, and thus were treated as potential candidate genes for plastic heterosis of fatty liver in hybrid ducks.

## Discussion

Understanding how organisms adapt to environmental changes through phenotypic plasticity remains a central challenge in evolutionary biology. Here, we applied comparative transcriptomic analyses to investigate how phenotypic plasticity and plastic heterosis manifest during fatty liver development under overfeeding conditions in Peking ducks, Muscovy ducks, and their reciprocal hybrids. We found that Muscovy ducks have preserved ancestral transcriptional plasticity in fatty acid oxidation pathways, whereas this plasticity has been lost in Peking ducks. In the hybrids, we observed substantial remodeling of parental allele-specific plasticity profiles. Remarkably, alleles originating from Peking ducks, which had lost plasticity in fatty acid oxidation, reacquired this ancestral plasticity, suggesting a mechanistic basis for plastic heterosis in hybrids. Together, our results demonstrate how phenotypic plasticity evolves in parental species and shapes plastic phenotypes in their interspecific hybrids.

**Fig. 6:**
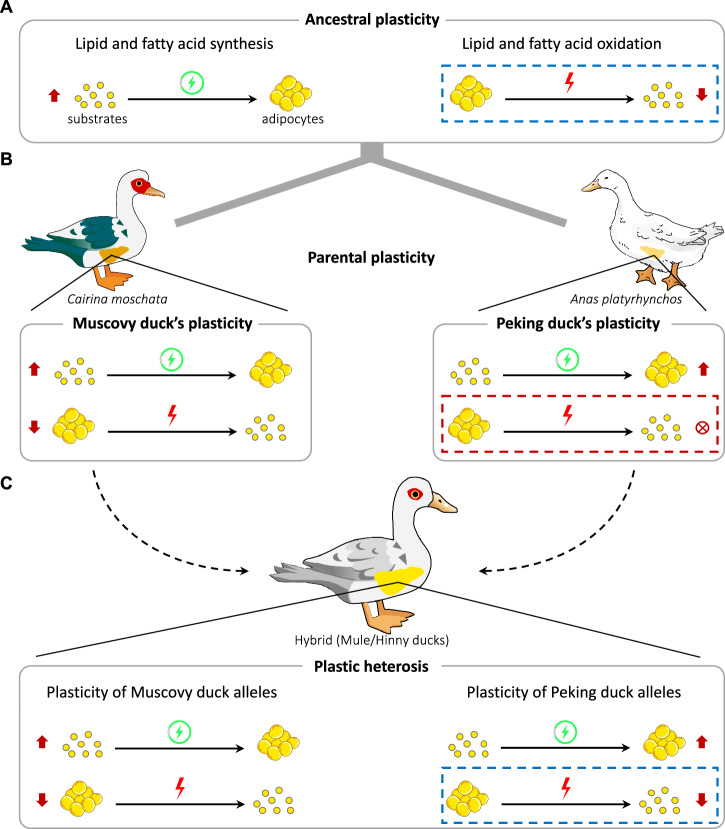
Schematic illustration of the potential mechanisms underlying plastic heterosis in hybrid ducks. **A** The LCA of Peking and Muscovy ducks developed transcriptional plasticity in fatty acid and lipid metabolism. This plasticity involved enhanced lipid and fatty acid biosynthesis and reduced lipid and fatty acid oxidation, serving as an ancient strategy to facilitate energy storage following overtaking. **B** Since divergence from the LCA, Muscovy ducks have largely retained this ancestral plasticity. In contrast, Peking ducks lost the plasticity associated with reduced lipid and fatty acid oxidation, leading to their lower susceptibility to fatty liver. **C** Interestingly, in hybrid ducks, Peking duck-origin alleles restore the lost plasticity, contributing to the manifestation of plastic heterosis in fatty liver development in response to overfeeding.

Fatty liver development is considered an ancient form of phenotypic plasticity in the LCA of waterfowl^28^. Consistent with this, we detected evidence of ancestral transcriptional plasticity in the LCA of Peking and Muscovy ducks, characterized by upregulated fatty acid synthesis and downregulated fatty acid oxidation (Fig. 6A), which together facilitate fatty liver formation. Moreover, gene expression plasticity generally aligns with the evolved divergences between Peking and Muscovy ducks, suggesting that the phenotypic plasticity associated with fatty liver development in waterfowl is adaptive. This finding is consistent with the notion that phenotypic plasticity can facilitate adaptive evolution^38–40^. Although both Peking and Muscovy ducks have retained ancestral plasticity, differences in the degree of phenotypic plasticity indicate divergent evolutionary trajectories. Specifically, Muscovy ducks have largely preserved ancestral plasticity, whereas Peking ducks have lost plasticity associated with fatty acid oxidation, resulting in reduced susceptibility to fatty liver (Fig. 6B). In particular, Muscovy ducks both exhibit higher overall transcriptomic evolutionary rates and maintain ancestral gene expression plasticity. Thus, retention of ancestral transcriptional plasticity and higher transcriptomic evolutionary rates are not mutually exclusive. These findings suggest that differences in transcriptional plasticity related to lipid and fatty acid catabolism may underlie the divergences in fatty liver susceptibility between these two duck species.

Based on the differential maintenance of ancestral plasticity in the context of fatty liver development, we infer that phenotypic plasticity represents an ancestral trait in Muscovy ducks while constituting a derived trait in Peking ducks. Notably, Muscovy ducks are tropical species native to Central and South America^41,42^. And the wild ancestors of Peking ducks (*Anas spp*.) also originated in South America^43,44^. The geographic proximity of the original ranges of these two species further supports this hypothesis. We therefore propose that ancestral plasticity was partially lost along the lineage leading to Peking ducks. This loss suggests that ancestral plasticity served as a foundational framework, from which modified or reduced forms of plasticity subsequently evolved. Consistent with the proposed “tinkering” model of phenotypic evolution^13,45,46^, species may evolve novel or modified forms of plasticity through a partial loss of pre-existing ancestral plasticity.

Mule and Hinny ducks, viable interspecific hybrids produced from crosses between Peking and Muscovy ducks, exhibit pronounced plastic heterosis in fatty liver development when subjected to overfeeding conditions^31,32^. Accordingly, we propose that the Peking-Muscovy-hybrid duck system serves as a robust and tractable model for investigating the genetic mechanisms underlying heterosis, particularly plastic heterosis, in animals. We provide evidence that Peking-origin alleles associated with fatty acid oxidation in hybrid ducks exhibited downregulation patterns similar to those of Muscovy-origin alleles (Fig. 6C). Intriguingly, the transcriptional plasticity of these alleles could be traced back to the LCA of Peking and Muscovy ducks, even though this plasticity was lost in Peking ducks. We propose that the restoration of ancestral plasticity in parental alleles within hybrids represents a mechanism contributing to plastic heterosis.

**Fig. 7:**
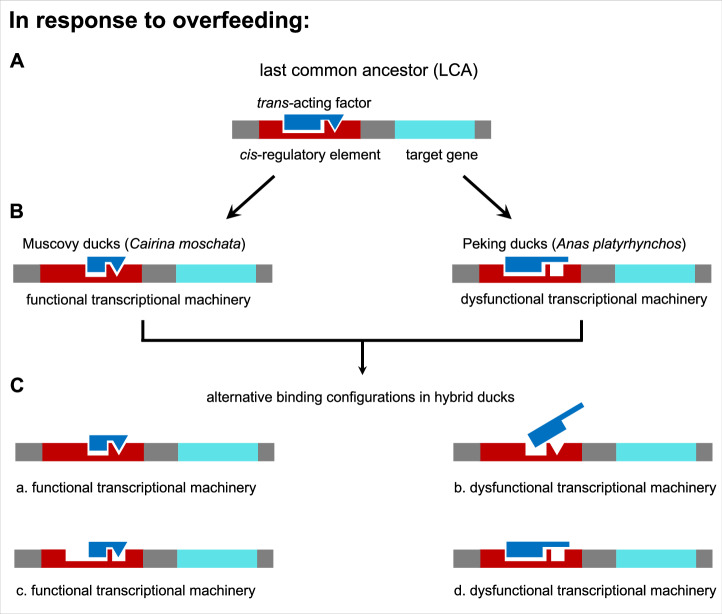
Proposed model for the restoration of ancestral transcriptional plasticity contributing to plastic heterosis in fatty liver of hybrid ducks. **A** In the LCA of Peking and Muscovy ducks, transcriptional plasticity in response to overconsumption likely relied on coordinated *cis*-*trans*-regulatory mechanisms that appropriately modulated fatty acid oxidation genes. **B** Following divergence, regulatory components might evolve asymmetrically in the two lineages. Muscovy ducks appear to have retained more of the ancestral regulatory architecture supporting plastic downregulation of fatty acid oxidation genes, whereas Peking ducks may have partially lost this plasticity due to weakened or absent *trans*-acting factors, possibly accompanied by alteration in *cis*-regulatory elements. **C** In hybrids, four distinct *cis*-*trans* combinations may arise. Notably, Muscovy-derived *trans*-acting factors can interact efficiently with Peking-derived *cis*-regulatory elements, redirecting Peking alleles toward Muscovy-like regulatory responses.

Directional shifts in parental allele expression have previously been observed in hybrids across tissues and environmental conditions, indicating that altered parental allele expression patterns may play distinct roles in heterosis^16^. Building on this, we demonstrate that shifts in plastic modes of parental alleles, such as P2M PPAs, also contribute prominently to heterosis. Integration of WGCNA results indicated that Peking- and Muscovy-derived alleles involved in lipid and fatty acid oxidation constitute a shared co-expression module and are coordinately regulated in response to overfeeding in hybrids. Such coordinated transcriptional relationships are absent or substantially weaker in Peking ducks. This suggests that hybridization can rewire transcriptional networks, enabling Peking-derived alleles to acquire new regulatory correlations in hybrids and thereby providing a potential mechanistic basis for the observed conversion of plasticity (Fig. 7). Emerging evidence suggests that 3D genome reorganization influences phenotypic plasticity in gene expression^47^, including during fatty liver development in Peking ducks^48^. This raises the possibility that environment-responsive reorganization of parental genomes in hybrids may underlie these altered plastic modes of parental alleles, providing further insight into the precise molecular mechanisms driving plastic heterosis.

Three primary non-additive models—dominance, overdominance, and epistasis—are widely recognized as explanations for heterosis^5^. Classical quantitative and population genetics studies attribute dominance as a major contributor to heterosis based on associations between loci and heterotic traits^6,7^. And dominance in gene expression has been implicated in the manifestation of heterosis^6,9^. If dominance in gene expression was the primary mechanism underlying plastic heterosis in hybrid ducks, genes showing parental dominance would be expected to play key roles in fatty liver development. However, we found that both Peking- and Muscovy-dominant genes exhibit limited enrichment in lipid metabolism-related pathways, indicating that plastic heterosis associated with fatty liver development in hybrid ducks is unlikely to be ascribed to parental dominance effects in gene expression alone. This finding implies that epistatic interactions may contribute substantially to plastic heterosis; however, evaluating these effects will require hundreds and even thousands of sample sizes to identify candidate heterotic QTLs^23,49^, warranting further investigation in future studies.

Our analysis of PPAs further characterized four candidate genes potentially involved in plastic heterosis. *ABCG5* encodes an ATP-binding cassette transporter that limits intestinal sterol absorption and promotes biliary sterol excretion. The disruption of *Abcg5* in mice can result in an accumulation of cholesterol in the liver after cholesterol feeding^50^. *ALDH1A1* encodes a rate-limiting enzyme that converts retinaldehyde to retinoic acid. Mice deficient in *Aldh1a1* exhibit suppressed triacylglycerol synthesis^51^. Conversely, knockdown experiments in chickens revealed an opposite effect of *ALDH1A1* on this process^52^. *APOB* encodes a key structural protein required for the assembly and secretion of very-low-density lipoproteins, which mediate lipid export from the liver^53^. Finally, *NADK2* encodes a mitochondrial nicotinamide adenine dinucleotide kinase. Decreased expression of *NADK2* in humans is significantly associated with the occurrence of non-alcoholic fatty liver disease, and depletion of the *Nadk2* gene in mice led to decreased fat oxidation^54^.

In summary, our results demonstrate that the evolved plasticity of gene expression observed in parental ducks is reshaped in their offspring during hybridization. The altered plasticity of parental alleles in hybrids contributes to plastic heterosis in fatty liver development. This work broadens our understanding of the evolution of gene expression plasticity across species and provides insights into the role of interspecific hybridization in shaping plasticity profiles of gene expression. It underscores the importance of parental allelic plasticity in heterotic traits and offers a perspective on the mechanisms underlying plastic heterosis.

## Materials and methods

### Animals and experiment design

Animals and experimental procedures were described previously^55^. Male Peking ducks (*Anas platyrhynchos*), Muscovy ducks (*Cairina moschata*), Mule ducks (♀ Peking × ♂ Muscovy), and Hinny ducks (♀ Muscovy × ♂ Peking) were reared under standard light and temperature conditions. From hatching to 6 weeks of age, all ducks were fed *ad libitum*. From 6 to 12 weeks of age, birds were maintained on a restricted diet appropriate for each genotype. At 12 weeks of age, ducks were assigned to one of two feeding treatments: (1) continued *ad libitum* feeding with the growing diet or (2) a 14-day overfeeding regimen using high-carbohydrate corn and corn meal. For liver sample collection, ducks were slaughtered 14 h after the final meal by electronarcosis, neck sectioning, and bleeding. Immediately thereafter, livers were weighed, sampled, rapidly frozen in liquid nitrogen, and stored at −80 °C. In total, ten biological replicates of liver tissue were collected for each genotype and feeding condition, except for Muscovy ducks under *ad libitum* feeding, for which nine biological replicates were available.

### Analysis of hepatic transcriptomic data of parental species

The RNA sequencing and data for Peking, Muscovy, and their crossbreed mule and Hinny ducks were reported before^31^. Considering Muscovy ducks under *ad libitum* feeding group contained nine replicates^32^, we maintained nine replicates of RNA-seq for liver samples across species and feeding conditions to avoid potential sampling bias (Supplementary Data 5). Raw hepatic transcriptomic data from Peking and Muscovy ducks were trimmed using Trimmomatic software^56^. The clean reads were aligned to respective reference genomes^35,57^ (Supplementary Data 6) separately using HISAT2^58^. Gene expressions were measured in manner of raw read counts by featureCounts^59^ and TPM (transcripts per million) by StringTie^60^. DEGs were identified using the DESeq2^61^ package with cutoffs: *p*adj < 0.01 and |log2FoldChange| > 1. In addition, the 13,417 one-to-one orthologous genes were retrieved from our previous study^35^, and transcriptomic analysis between Peking and Muscovy ducks was conducted using these orthologous genes.

### Inferences of ancestral plasticity between parental species

All three biological replicates of liver samples from Tianfu meet goose breed under *ad libitum* and overfed conditions^62^ (Supplementary Data 5) were included in our analysis. The RNA-seq reads were first trimmed by Trimmomatic. We employed HISAT2 to map clean reads to the goose reference genome^63^ (Supplementary Data 6). TPM values of gene expression were calculated by StringTie. To identify one-to-one orthologs among Peking ducks, Muscovy ducks, and geese, we applied the reciprocal best hit approach^64^. The longest transcript for each Peking duck gene was extracted and used as the reference for reciprocal BLASTN searches against transcript sequences of Peking and Muscovy ducks, using the parameters： -num_threads 10 -evalue 1e-20 -outfmt 6. Gene pairs that were mutual best hits were retained as one-to-one orthologs. Using this approach, we identified 11,682 one-to-one orthologous genes among Peking ducks, Muscovy ducks, and geese (Supplementary Data 7).

We utilized TPM values of gene expression as inputs for subsequent bioinformatics analysis, with the goose hepatic transcriptome serving as the outgroup. A rooted and ultrametric tree including Peking duck, Muscovy duck, and goose lineages was extracted from our previous study^35^ and is publicly available on GitHub (https://github.com/qiuyixmm/Plastic-Heterosis-Paper). To compare the evolutionary rate of transcriptomes between Peking and Muscovy ducks, we used CAGEE software^36^ to estimate branch-specific evolutionary rate parameters (*σ*²) along the input phylogeny. Specifically, CAGEE models the evolution of gene expression as a continuous quantitative trait along phylogenetic branches, providing rate parameters (*σ*²) that describe the variance in expression change per unit evolutionary time. Expression matrices corresponding to *ad libitum* and overfed conditions were analyzed in separate CAGEE runs, allowing *σ*² to be estimated independently and ancestral expression levels to be inferred separately for each feeding condition. This software was also utilized to infer gene expression levels of the LCA in Peking and Muscovy ducks separately for the two feeding conditions. To evaluate the robustness of ancestral plastic gene identification with respect to expression quantification, we re-estimated ancestral gene expression levels using TMM-normalized CPM (Trimmed Mean of M-values–normalized counts per million) generated with the edgeR package^65^. GO enrichment performed on TMM-normalized CPM-based ancestral plastic genes yielded results consistent with those inferred from TPM inputs (Supplementary Fig. 4 and Supplementary Data 3).

### Classification of plasticity modes in gene expression of parental species

We utilized the previously established bioinformatic pipeline^34^ to access adaptive or nonadaptive plasticity for each gene in gene expression. We calculated gene expression differences between Peking and Muscovy using a *t*-statistic and validated their significances via a nonparametric sign test (*n* = 250). The concordantly differentially expressed (CDE) genes between Peking and Muscovy ducks were identified as those exhibiting gene expression differences independent of feeding conditions. For CDE genes, gene expression plasticity in Peking ducks was quantified between fed *ad libitum* and overfed conditions, and evolutionary divergence in gene expression was quantified between Peking and Muscovy ducks under overfed conditions. We then calculated the Spearman’s rank correlation between expression plasticity and evolutionary divergences and compared it to the distribution from 1000 random permutations. Genes with plasticity in the same direction as evolutionary divergence were classified as adaptive; those with plasticity in the opposite direction were considered nonadaptive.

### Analysis of fatty liver weights for parental and hybrid ducks

To validate the reported plastic heterosis in fatty liver development of hybrid ducks, we extracted liver weight data from previous studies^31^ for both parental species and their hybrids under two feeding conditions. This cohort of ducks was also subjected to the transcriptomic study referenced in ref. ^32^. Differences in liver weights among groups were assessed using a *t*-test. The percentage of heterosis (*H*%) was defined as the increase in performance of the offspring relative to the average of the parents, and was calculated by the following formula:

$$H \% =\frac{F{1}_{{\mbox{avg.}}}-0.5\times ({M}_{{\mbox{avg.}}}+{P}_{{\mbox{avg.}}})}{0.5\times ({M}_{{\mbox{avg.}}}+{P}_{{\mbox{avg.}}})}\times 100 \%$$

where avg. denotes the average value. The significance of *H*% was tested using *t*-statistics.

### Analysis of hepatic transcriptomic data of hybrids

Whole-genome alignment using MUMmer^66^ estimated an average genomic sequence divergence of approximately 5.6% between Peking and Muscovy ducks. This level of divergence greatly exceeds typical within-species polymorphism and precludes reliable allelic read assignment based on single-nucleotide polymorphisms (SNPs) in interspecific hybrids. Consistent with this, cross-species read mapping showed markedly reduced mappability when Peking RNA-seq reads were aligned to the Muscovy genome and vice versa (Supplementary Table 3). Together, these substantial genomic and transcriptomic divergences indicate that canonical SNP-aware approaches being developed to correct reference-allele bias within species are not appropriate for this interspecific system.

To quantify allele- and gene-level expression in hybrids, we therefore constructed a pseudo-haplotype-resolved hybrid genome by concatenating the Peking and Muscovy genome assemblies (Supplementary Fig. 12). A total of 13,417 one-to-one orthologous genes were identified and designated as parental alleles within the hybrid genome. RNA-seq data for Mule and Hinny ducks were obtained from the same study^32^. Clean reads were aligned to the pseudo-haplotype-resolved hybrid genome using HISAT2 with default parameters, and multi-mapping and unpaired reads were removed using SAMtools^67^. Allelic read counts were quantified using featureCounts^59^ (-T 5 -J -p -B -C -t exon -g gene_id) and summed to represent gene-level expression for alleles of Peking and Muscovy origin. Differentially expressed alleles between *ad libitum* and overfed conditions were identified separately for Peking- and Muscovy-derived alleles using DESeq2 (*p*adj < 0.01, |log₂FoldChange| > 1). To assess the transcriptional similarity between parental and hybrid ducks, we calculated Kendall’s rank correlation using log-transformed, normalized read counts from the DESeq2 package.

To evaluate the potential impact of read-mapping artifacts on allelic expression quantification, we conducted a simulation-based assessment (Supplementary Fig. 12). Briefly, 50% of RNA-seq reads from Peking and Muscovy ducks were randomly downsampled, labeled by parental origin, and merged to generate pseudo-hybrid datasets under both feeding conditions. These downsampled reads were aligned to the pseudo-haplotype-resolved hybrid genome using the same mapping and filtering criteria. Gene-level assignments were subsequently generated using featureCounts (parameters: -R CORE -T 5 -J -p -B -C -t exon -g gene_id). Read-pair assignments to genes on each haplotype were compared with the known parental origin of each read to show that the majority of reads were correctly assigned to their parental haplotypes and genes, with low misassignment rates (Supplementary Data 8). We further employed DESeq2 as above to show simulated pseudo-hybrids recapitulated parental expression responses to feeding condition changes (Supplementary Fig. 13). These results indicated that our pipeline of transcriptomic data analysis for hybrid ducks was robust to potential read mapping artifacts.

For gene co-transcription analysis, WGCNA results for parental ducks were obtained from our previous study^35^. In contrast, for hybrid ducks, WGCNA was conducted using allelic expression levels to capture co-expression patterns of parental alleles within the hybrid genome.

### Classification of inheritance modes of gene expression in hybrid ducks

We adopted previously established criteria^14^ to classify the inheritance of gene expression into different modes in hybrid ducks. Briefly, *conserved* genes were defined as those exhibiting minimal expression differences between hybrids and both parental ducks. *Additive* genes were identified as those with expression levels in hybrids greater than one parental species but lower than the other. *Muscovy-dominant* (or *Peking-dominant*) genes were identified as those displaying minimal expression differences between hybrids and Muscovy (or Peking) ducks, but significant differences between hybrids and the alternate parental species. *Overdominant* genes were identified as those whose expression levels in hybrids exceeded those of both parental species, while *underdominant* genes were those whose expression levels in hybrids were lower than those of both parental species. Genes were considered to exhibit different expression levels if the total expression in hybrids deviated by more than 1.25-fold from that of either parent.

### Functional enrichment analysis

Gene Ontology (GO) enrichment analyses of genes or alleles in this study were conducted using clusterProfiler^68^. GO items with *p* value < 0.05 and *q* value < 0.05 were regarded as a significant enrichment. GSEA was performed using the gseGO function in the clusterProfiler package. The GSEA results were filtered with parameters: *p* value < 0.05 and *q* value < 0.25.

### Statistics and reproducibility

Statistical methods and parameters were described in the “Materials and methods” section. All statistical analyses were performed using R version 4.3.0.

### Ethics statement

This study used data derived from publicly available databases and did not involve live vertebrate animals or human participants. All analyses were performed in accordance with relevant guidelines and regulations. Therefore, ethics approval and informed consent were not applicable to this study. For clarity, sample information is summarized from their original publications as follows.

Duck samples were originally generated in previous studies. Briefly, ducks of four genotypes were provided by Grimaud (Roussay, France), and liver samples were collected in accordance with French national guidelines for animal care and use, as described in the original study^55^. These liver samples were subsequently reused for RNA extraction and sequencing in later studies^31,32^. For goose samples, Tianfu geese were obtained from the Sichuan Agricultural University Waterfowl Breeding Farm (Sichuan, China), as described in the original publication^62^. Liver samples were collected for RNA extraction and sequencing with approval from the Sichuan Agricultural University Animal Welfare Committee.

## Supplementary information

Supplementary Information

Description of Additional Supplementary Files

Supplementary Data 1

Supplementary Data 2

Supplementary Data 3

Supplementary Data 4

Supplementary Data 5

Supplementary Data 6

Supplementary Data 7

Supplementary Data 8

Reporting_summary

## Data Availability

All data used in this study are publicly available. Transcriptomic data for Peking, Muscovy, Mule, and Hinny ducks are available in the NCBI Sequence Read Archive under accession number SRP144764. Transcriptomic data for geese are available in the Gene Expression Omnibus under accession number GSE119421. Source data for all figures in the main text and supplementary materials are available via Zenodo (10.5281/zenodo.19151283)^69^. Any remaining related information can be obtained from the corresponding author upon reasonable request.

## Code availability

The scripts used to perform the analyses and generate results in this study are publicly available and have been deposited in GitHub (https://github.com/qiuyixmm/Plastic-Heterosis-Paper) and Zenodo (10.5281/zenodo.19151283)^69^.

## References

[1] Barton, N. H. The role of hybridization in evolution. *Mol. Ecol.* **10**, 551–568 (2001). 11298968 10.1046/j.1365-294x.2001.01216.x

[2] Lippman, Z. B. & Zamir, D. Heterosis: revisiting the magic. *Trends Genet.* **23**, 60–66 (2007). 17188398 10.1016/j.tig.2006.12.006

[3] Thompson, K. A. & Schluter, D. Heterosis counteracts hybrid breakdown to forestall speciation by parallel natural selection. *Proc. Biol. Sci.* **289**, 20220422 (2022). 35506223 10.1098/rspb.2022.0422 PMC9065978

[4] Birchler, J. A., Yao, H. & Chudalayandi, S. Unraveling the genetic basis of hybrid vigor. *Proc. Natl. Acad. Sci. USA* **103**, 12957–12958 (2006). 16938847 10.1073/pnas.0605627103 PMC1559732

[5] Liu, W., Zhang, Y., He, H., He, G. & Deng, X. W. From hybrid genomes to heterotic trait output: challenges and opportunities. *Curr. Opin. Plant Biol.* **66**, 102193 (2022). 35219140 10.1016/j.pbi.2022.102193

[6] Cui, L. et al. Dominance is common in mammals and is associated with trans-acting gene expression and alternative splicing. *Genome Biol.* **24**, 215 (2023). 37773188 10.1186/s13059-023-03060-2 PMC10540365

[7] Zeng, Z. B., Gesteira, G. S., Mo, L., Xiao, Y. & Yan, J. A theory of heterosis. *Genetics* **230**, 10.1093/genetics/iyaf045 (2025). 10.1093/genetics/iyaf045 40101147

[8] Tirosh, I., Reikhav, S., Levy, A. A. & Barkai, N. A yeast hybrid provides insight into the evolution of gene expression regulation. *Science* **324**, 659–662 (2009). 19407207 10.1126/science.1169766

[9] Liu, W., He, G. & Deng, X. W. Biological pathway expression complementation contributes to biomass heterosis in Arabidopsis. *Proc. Natl. Acad. Sci. USA* **118**, 10.1073/pnas.2023278118 (2021). 10.1073/pnas.2023278118 PMC8072403 33846256

[10] Buggs, R. J. et al. Transcriptomic shock generates evolutionary novelty in a newly formed, natural allopolyploid plant. *Curr. Biol.* **21**, 551–556 (2011). 21419627 10.1016/j.cub.2011.02.016

[11] Quan, C. et al. Transcriptome shock in interspecific F1 allotriploid hybrids between Brassica species. *J. Exp. Bot.* **73**, 2336–2353 (2022). 35139197 10.1093/jxb/erac047

[12] Herbst, R. H. et al. Heterosis as a consequence of regulatory incompatibility. *BMC Biol.* **15**, 38 (2017). 28494792 10.1186/s12915-017-0373-7 PMC5426048

[13] Wray, G. A. The evolutionary significance of cis-regulatory mutations. *Nat. Rev. Genet.* **8**, 206–216 (2007). 17304246 10.1038/nrg2063

[14] McManus, C. J. et al. Regulatory divergence in Drosophila revealed by mRNA-seq. *Genome Res.* **20**, 816–825 (2010). 20354124 10.1101/gr.102491.109 PMC2877578

[15] Ma, Y. et al. Identification of allele-specific expression genes associated with maize heterosis. *Agronomy* **13**, 10.3390/agronomy13112722 (2023).

[16] Shao, L. et al. Patterns of genome-wide allele-specific expression in hybrid rice and the implications on the genetic basis of heterosis. *Proc. Natl. Acad. Sci. USA* **116**, 5653–5658 (2019). 30833384 10.1073/pnas.1820513116 PMC6431163

[17] Zhan, W. et al. Natural variations of heterosis-related allele-specific expression genes in promoter regions lead to allele-specific expression in maize. *BMC Genom.* **25**, 476 (2024). 10.1186/s12864-024-10395-y PMC11092226 38745122

[18] Wang, H. et al. From heterosis to outbreeding depression: genotype-by-environment interaction shifts hybrid fitness in opposite directions. *Genetics*. 10.1093/genetics/iyae090 (2024). 10.1093/genetics/iyae090 38809057

[19] Payne, J. L. & Wagner, A. The causes of evolvability and their evolution. *Nat. Rev. Genet.* **20**, 24–38 (2019). 30385867 10.1038/s41576-018-0069-z

[20] Sommer, R. J. Phenotypic plasticity: from theory and genetics to current and future challenges. *Genetics* **215**, 1–13 (2020). 32371438 10.1534/genetics.120.303163 PMC7198268

[21] Kelly, S. A., Panhuis, T. M. & Stoehr, A. M. Phenotypic plasticity: molecular mechanisms and adaptive significance. *Compr. Physiol.* **2**, 1417–1439 (2012). 23798305 10.1002/cphy.c110008

[22] Bernatchez, L., Ferchaud, A. L., Berger, C. S., Venney, C. J. & Xuereb, A. Genomics for monitoring and understanding species responses to global climate change. *Nat. Rev. Genet.* **25**, 165–183 (2024). 37863940 10.1038/s41576-023-00657-y

[23] Liu, N., Du, Y., Warburton, M. L., Xiao, Y. & Yan, J. Phenotypic plasticity contributes to maize adaptation and heterosis. *Mol. Biol. Evol.* **38**, 1262–1275 (2021). 33212480 10.1093/molbev/msaa283 PMC8480182

[24] Morris, M. R., Fraser, D. J., Eddington, J. & Hutchings, J. A. Hybridization effects on phenotypic plasticity: experimental compensatory growth in farmed-wild Atlantic salmon. *Evol. Appl.* **4**, 444–458 (2011). 25567994 10.1111/j.1752-4571.2010.00159.x PMC3352526

[25] Ballinger, M. A., Mack, K. L., Durkin, S. M., Riddell, E. A. & Nachman, M. W. Environmentally robust cis-regulatory changes underlie rapid climatic adaptation. *Proc. Natl. Acad. Sci. USA* **120**, e2214614120 (2023). 37725649 10.1073/pnas.2214614120 PMC10523592

[26] Vlkova, M. & Silander, O. K. Gene regulation in Escherichia coli is commonly selected for both high plasticity and low noise. *Nat. Ecol. Evol.* **6**, 1165–1179 (2022). 35726087 10.1038/s41559-022-01783-2

[27] Bei, C. et al. Genetically encoded transcriptional plasticity underlies stress adaptation in Mycobacterium tuberculosis. *Nat. Commun.* **15**, 3088 (2024). 38600064 10.1038/s41467-024-47410-5 PMC11006872

[28] Hermier, D. et al. Plasma lipoproteins and liver lipids in two breeds of geese with different susceptibility to hepatic steatosis: changes induced by development and force-feeding. *Lipids* **26**, 331–339 (1991). 1895877 10.1007/BF02537194

[29] Jiang, F. et al. A chromosome-level genome assembly of Cairina moschata and comparative genomic analyses. *BMC Genom.* **22**, 581 (2021). 10.1186/s12864-021-07897-4 PMC8325232 34330207

[30] B. Islam, F. et al. Male hybrid sterility in the Mule duck is associated with meiotic arrest in primary spermatocytes. * J. Poult. Sci.* **50**, 311–320 (2013).

[31] Herault, F., Baeza, E. & Diot, C. RNA-Seq transcriptome data of the liver of common Pekin, Muscovy, Mule and Hinny ducks fed ad libitum or overfed. *Data Brief* **39**, 107516 (2021). 34765707 10.1016/j.dib.2021.107516 PMC8572865

[32] Herault, F. et al. RNA-seq analysis of hepatic gene expression of common Pekin, Muscovy, Mule and Hinny ducks fed ad libitum or overfed. *BMC Genom.* **20**, 13 (2019). 10.1186/s12864-018-5415-1 PMC6323773 30616512

[33] Chapuis, H. et al. Genetic parameters of feeding behaviour traits in ducks bred for foie gras production. *Animal* **18**, 101234 (2024). 39059119 10.1016/j.animal.2024.101234

[34] Ghalambor, C. K. et al. Non-adaptive plasticity potentiates rapid adaptive evolution of gene expression in nature. *Nature* **525**, 372–375 (2015). 26331546 10.1038/nature15256

[35] Xu, M. M. et al. Chromosome-level genome assembly of the Muscovy duck provides insight into fatty liver susceptibility. *Genomics* **114**, 110518 (2022). 36347326 10.1016/j.ygeno.2022.110518

[36] Bertram, J. et al. CAGEE: Computational Analysis of Gene Expression Evolution. *Mol. Biol. Evol.* **40**, 10.1093/molbev/msad106 (2023). 10.1093/molbev/msad106 PMC10195155 37158385

[37] Langfelder, P. & Horvath, S. WGCNA: an R package for weighted correlation network analysis. *BMC Bioinform.* **9**, 559 (2008). 10.1186/1471-2105-9-559 PMC2631488 19114008

[38] Bilandzija, H. et al. Phenotypic plasticity as a mechanism of cave colonization and adaptation. *eLife* **9**, 10.7554/eLife.51830 (2020). 10.7554/eLife.51830 PMC7173965 32314737

[39] Levis, N. A., Isdaner, A. J. & Pfennig, D. W. Morphological novelty emerges from pre-existing phenotypic plasticity. *Nat. Ecol. Evol.* **2**, 1289–1297 (2018). 29988161 10.1038/s41559-018-0601-8

[40] Xue, B. et al. Phenotypic plasticity co-varies with elevational range in two avian species of elevational migrants in the Himalayas. *Nat. Commun.* **16**, 5316 (2025). 40533451 10.1038/s41467-025-60770-w PMC12177054

[41] Stahl, P. W. An exploratory osteological study of the Muscovy duck (Cairina moschata) (Aves: Anatidae) with implications for neotropical archaeology. *J. Archaeol. Sci.* **32**, 915–929 (2005).

[42] Rodenburg, T. B. et al. Welfare of ducks in European duck husbandry systems. *World’s Poult. Sci. J.* **61**, 633–646 (2019).

[43] Livezey, B. C. A phylogenetic analysis and classification of recent dabbling ducks (Tribe Anatini) based on comparative morphology. *Auk* **108**, 471–507 (1991).

[44] Zhang, Z., Ai, H. & Huang, L. Whole-genome sequences restore the original classification of dabbling ducks (genus Anas). *Genet Sel. Evol.* **56**, 37 (2024). 38741064 10.1186/s12711-024-00904-8 PMC11089735

[45] Jacob, F. Evolution and tinkering. *Science* **196**, 1161–1166 (1977). 860134 10.1126/science.860134

[46] Rockman, M. V. & Stern, D. L. Tinker where the tinkering’s good. *Trends Genet.* **24**, 317–319 (2008). 18514359 10.1016/j.tig.2008.04.003 PMC2887039

[47] Li, A. et al. Genome architecture and selective signals compensatorily shape plastic response to a new environment. *Innovation* **4**, 100464 (2023). 37485076 10.1016/j.xinn.2023.100464 PMC10362523

[48] Ning, M. et al. Multiscale 3D genome organization underlies duck fatty liver with no adipose inflammation or serious injury. *Int. J. Biol. Macromol.* **271**, 132452 (2024). 38777007 10.1016/j.ijbiomac.2024.132452

[49] Torgeman, S. & Zamir, D. Epistatic QTLs for yield heterosis in tomato. *Proc. Natl. Acad. Sci. USA* **120**, e2205787119 (2023). 36972451 10.1073/pnas.2205787119 PMC10083602

[50] Yu, L. et al. Disruption of Abcg5 and Abcg8 in mice reveals their crucial role in biliary cholesterol secretion. *Proc. Natl. Acad. Sci. USA* **99**, 16237–16242 (2002). 12444248 10.1073/pnas.252582399 PMC138595

[51] Kiefer, F. W. et al. Retinaldehyde dehydrogenase 1 coordinates hepatic gluconeogenesis and lipid metabolism. *Endocrinology* **153**, 3089–3099 (2012). 22555438 10.1210/en.2011-2104 PMC3380298

[52] Zhang, J. et al. ALDH1A1 inhibits chicken preadipocytes’ proliferation and differentiation via the PPARgamma pathway in vitro and in vivo. *Int. J. Mol. Sci.* **21**, 10.3390/ijms21093150 (2020). 10.3390/ijms21093150 PMC7246604 32365706

[53] Choi, S. H. & Ginsberg, H. N. Increased very low density lipoprotein (VLDL) secretion, hepatic steatosis, and insulin resistance. *Trends Endocrinol. Metab.* **22**, 353–363 (2011). 21616678 10.1016/j.tem.2011.04.007 PMC3163828

[54] Kim, H. et al. The mitochondrial NAD kinase functions as a major metabolic regulator upon increased energy demand. *Mol. Metab.* **64**, 101562 (2022). 35944895 10.1016/j.molmet.2022.101562 PMC9403569

[55] Chartrin, P. et al. Does overfeeding enhance genotype effects on liver ability for lipogenesis and lipid secretion in ducks? *Comp. Biochem. Physiol. A Mol. Integr. Physiol.* **145**, 390–396 (2006). 16963298 10.1016/j.cbpa.2006.07.014

[56] Bolger, A. M., Lohse, M. & Usadel, B. Trimmomatic: a flexible trimmer for Illumina sequence data. *Bioinformatics* **30**, 2114–2120 (2014). 24695404 10.1093/bioinformatics/btu170 PMC4103590

[57] Li, J. et al. A new duck genome reveals conserved and convergently evolved chromosome architectures of birds and mammals. *Gigascience* **10**, 10.1093/gigascience/giaa142 (2021). 10.1093/gigascience/giaa142 PMC7787181 33406261

[58] Kim, D., Paggi, J. M., Park, C., Bennett, C. & Salzberg, S. L. Graph-based genome alignment and genotyping with HISAT2 and HISAT-genotype. *Nat. Biotechnol.* **37**, 907–915 (2019). 31375807 10.1038/s41587-019-0201-4 PMC7605509

[59] Liao, Y., Smyth, G. K. & Shi, W. featureCounts: an efficient general purpose program for assigning sequence reads to genomic features. *Bioinformatics* **30**, 923–930 (2014). 24227677 10.1093/bioinformatics/btt656

[60] Pertea, M., Kim, D., Pertea, G. M., Leek, J. T. & Salzberg, S. L. Transcript-level expression analysis of RNA-seq experiments with HISAT, StringTie and Ballgown. *Nat. Protoc.* **11**, 1650–1667 (2016). 27560171 10.1038/nprot.2016.095 PMC5032908

[61] Love, M. I., Huber, W. & Anders, S. Moderated estimation of fold change and dispersion for RNA-seq data with DESeq2. *Genome Biol.* **15**, 550 (2014). 25516281 10.1186/s13059-014-0550-8 PMC4302049

[62] Wang, G. et al. Transcriptomic analysis between Normal and high-intake feeding geese provides insight into adipose deposition and susceptibility to fatty liver in migratory birds. *BMC Genom.* **20**, 372 (2019). 10.1186/s12864-019-5765-3 PMC6518675 31088359

[63] Li, Y. et al. Pacific Biosciences assembly with Hi-C mapping generates an improved, chromosome-level goose genome. *Gigascience* **9**, 10.1093/gigascience/giaa114 (2020). 10.1093/gigascience/giaa114 PMC7585555 33099628

[64] Wall, D. P., Fraser, H. B. & Hirsh, A. E. Detecting putative orthologs. *Bioinformatics* **19**, 1710–1711 (2003). 15593400 10.1093/bioinformatics/btg213

[65] Robinson, M. D., McCarthy, D. J. & Smyth, G. K. edgeR: a bioconductor package for differential expression analysis of digital gene expression data. *Bioinformatics* **26**, 139–140 (2010). 19910308 10.1093/bioinformatics/btp616 PMC2796818

[66] Kurtz, S. et al. Versatile and open software for comparing large genomes. *Genome Biol.* **5**, R12 (2004). 14759262 10.1186/gb-2004-5-2-r12 PMC395750

[67] Li, H. et al. The Sequence Alignment/Map format and SAMtools. *Bioinformatics* **25**, 2078–2079 (2009). 19505943 10.1093/bioinformatics/btp352 PMC2723002

[68] Wu, T. et al. clusterProfiler 4.0: a universal enrichment tool for interpreting omics data. *Innovation* **2**, 100141 (2021). 34557778 10.1016/j.xinn.2021.100141 PMC8454663

[69] Xu. Restoration of parental ancestral plasticity contributes to plastic heterosis in the fatty liver of hybrid ducks. Zenodo, 10.5281/zenodo.19151283 (2026). 10.1038/s42003-026-10049-7 PMC13266055 41981108

