# Supplementary material for: Restoration of ancestral transcriptional plasticity contributes to plastic heterosis in fatty liver of hybrid ducks
Source: Commun Biol. 2026 Apr 14;9:803. doi: 10.1038/s42003-026-10049-7 (PMC13266055; doi:10.1038/s42003-026-10049-7)
Supplement: Supplementary file 1 — Supplementary Information [file 42003_2026_10049_MOESM1_ESM.pdf]

Supplementary Figures

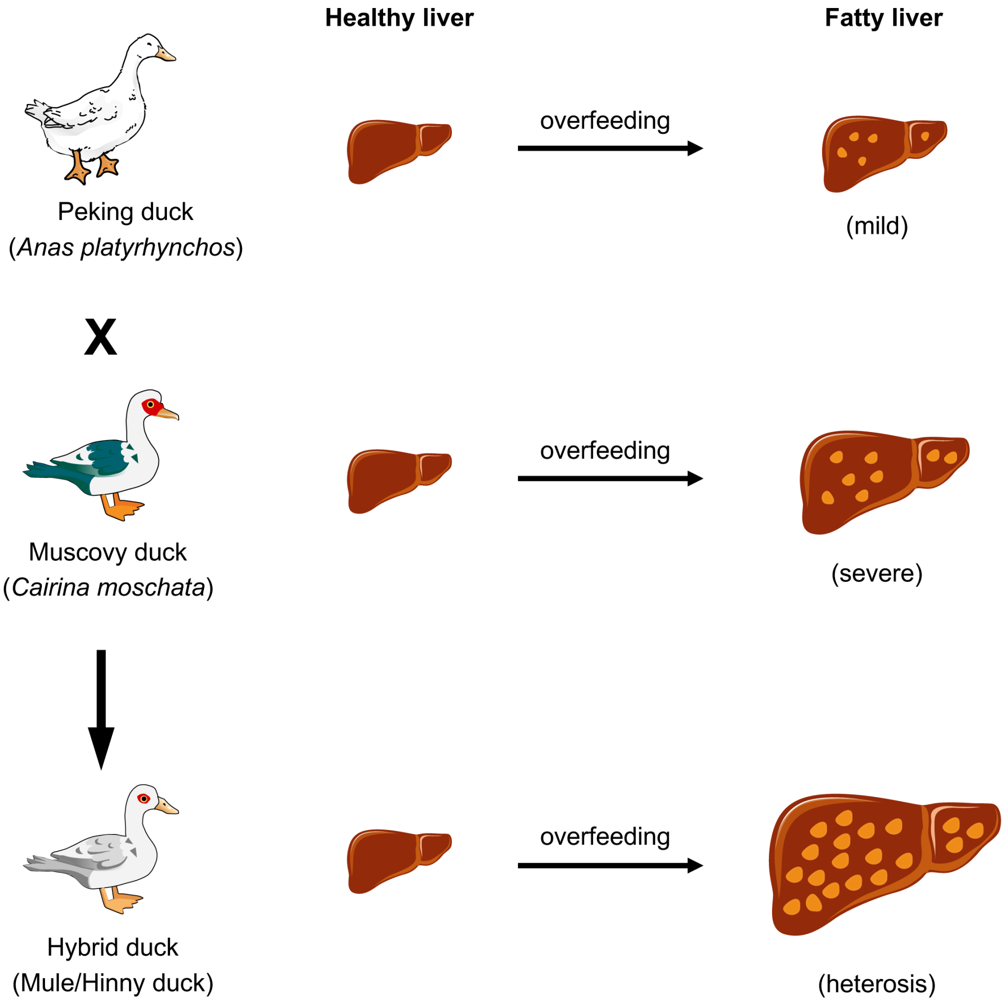

Supplementary Figure 1. The diverse susceptibility to fatty liver in Peking, Muscovy ducks, and their hybrids. A schematic illustration depicting the varying degrees of fatty liver development in Muscovy, Peking, and hybrid ducks. Upon overfeeding, Muscovy ducks exhibit severe fatty liver, whereas Peking ducks develop only mild fatty liver. Mule and Hinny ducks, the interspecific hybrids, display plastic heterosis with respect to fatty liver development.

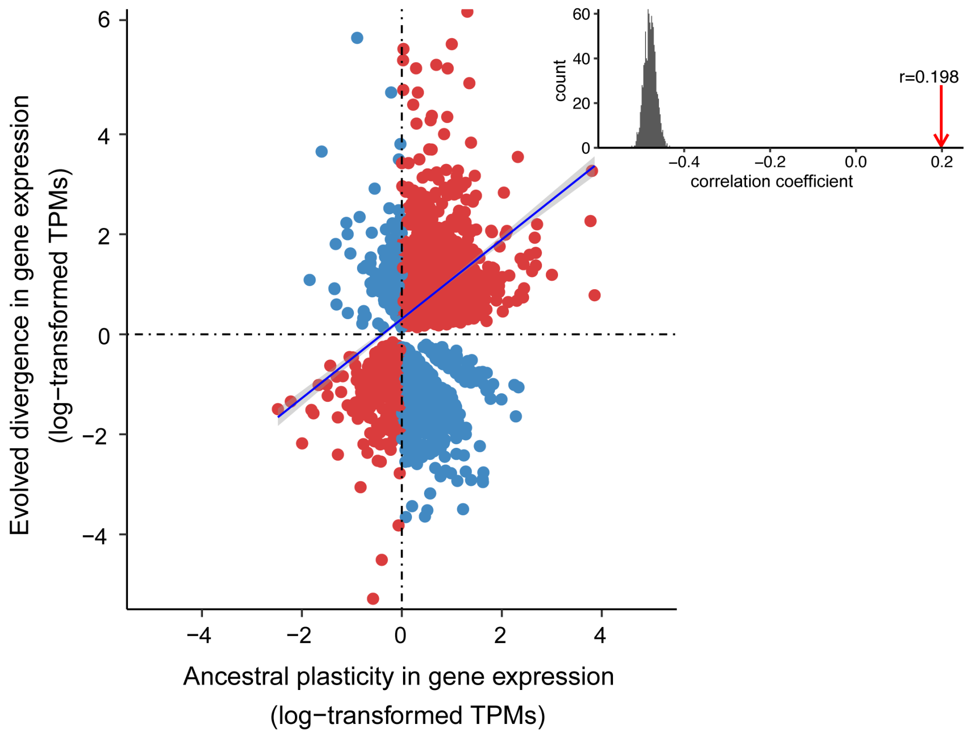

Supplementary Figure 2. A scatter plot illustrates the correlation between plasticity and evolved divergences in gene expression. Blue points represent transcripts with non-adaptive plasticity, while red points indicate those with adaptive plasticity. The upper right plot displays the distribution of spearman rank correlations from 1,000 permuted correlation values, with the red arrow highlighting the observed correlation.

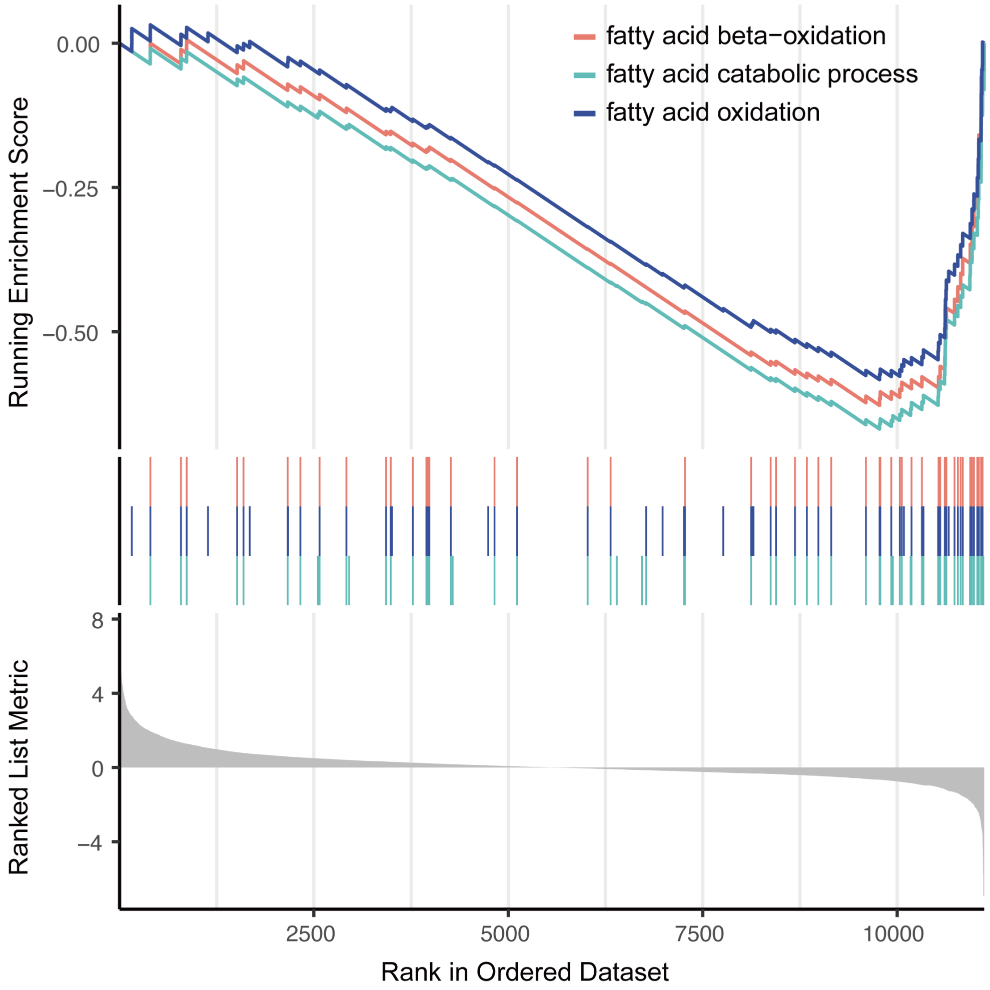

Supplementary Figure 3. GSEA shows that the lipid catabolism pathway was downregulated in livers of overfed Muscovy ducks.

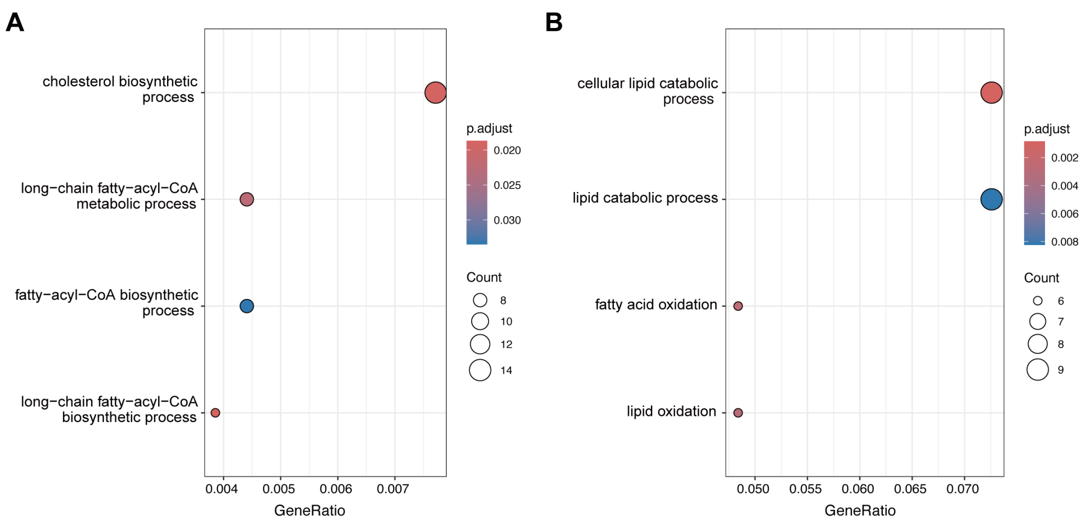

Supplementary Figure 4. Dot plots showing selected enriched GO terms for ancestral plastic genes. (**A**) Upregulated ancestral plastic genes. (**B**) Downregulated ancestral plastic genes.

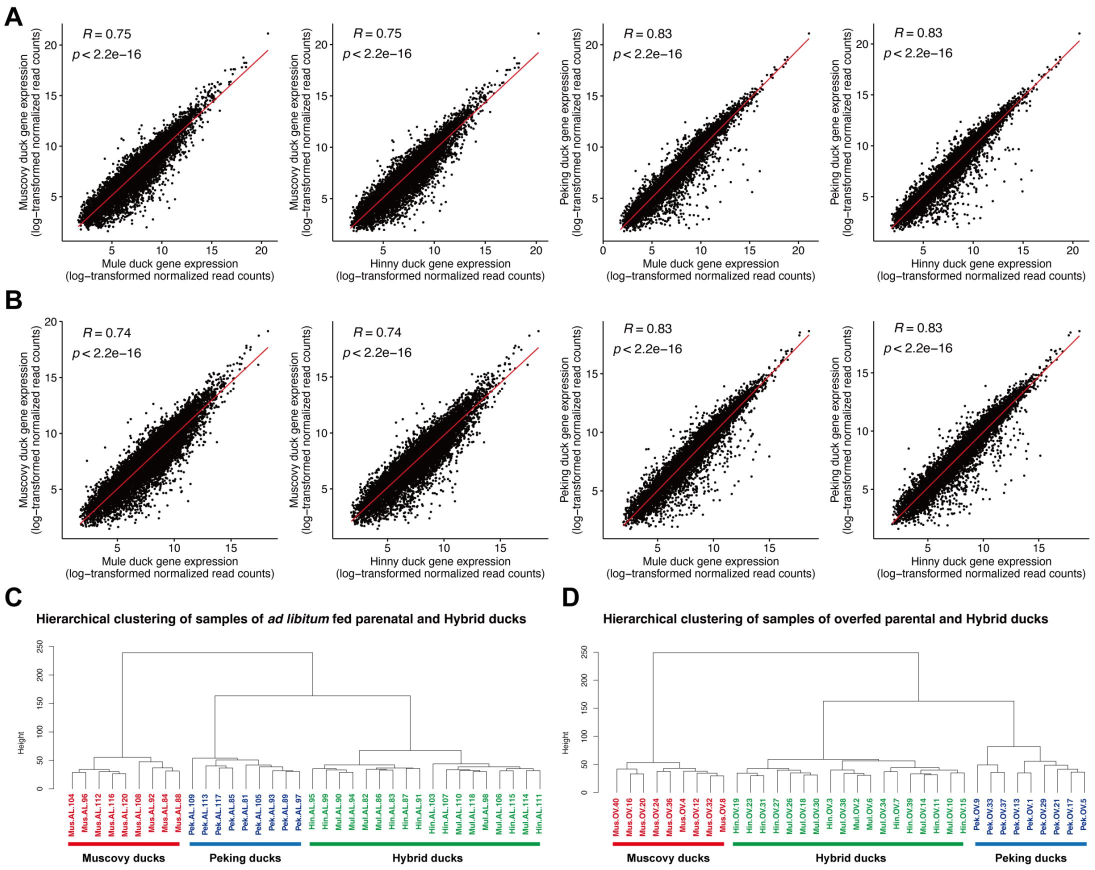

Supplementary Figure 5. Transcriptional similarity between parental and hybrid ducks under *ad libitum* and overfed conditions. (**A-B**) Pairwise correlations of global gene expression profiles between parental and hybrid ducks under *ad libitum* (**A**) and overfed (**B**) conditions. Transcriptional similarity was quantified using Kendall's rank correlation coefficient. Correlations were calculated based on log-transformed, normalized read counts from DESeq2, averaged across biological replicates for each species or hybrid under each feeding condition. (**C-D**) Hierarchical clustering of samples based on global gene expression profiles under *ad libitum* (**C**) and overfed (**D**) conditions.

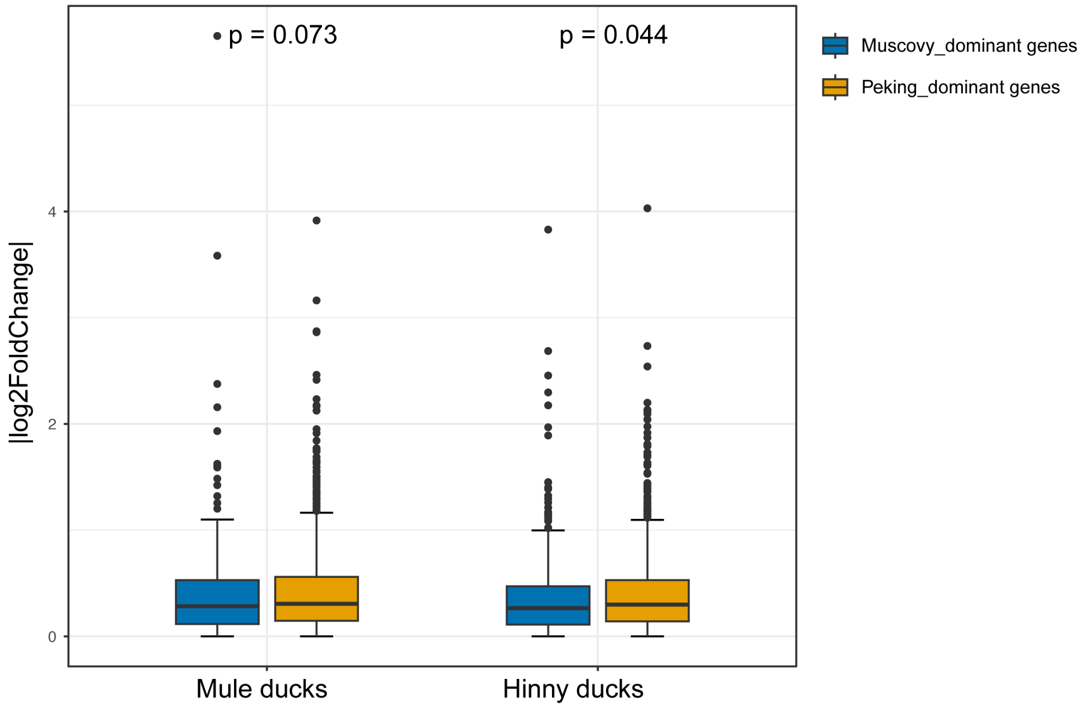

Supplementary Figure 6. Effect size of parent-dominant genes in hybrids. Effect sizes were measured as absolute $\log_2$ fold changes for Muscovy- and Peking-dominant genes in Mule (left) and Hinny (right) ducks. P-values were calculated using the Wilcoxon rank-sum test and are shown at the top of each panel. The error bars indicate the whiskers of the boxplot, defined as the most extreme data points within 1.5 × the interquartile range (IQR) from the lower (Q1) and upper (Q3) quartiles.

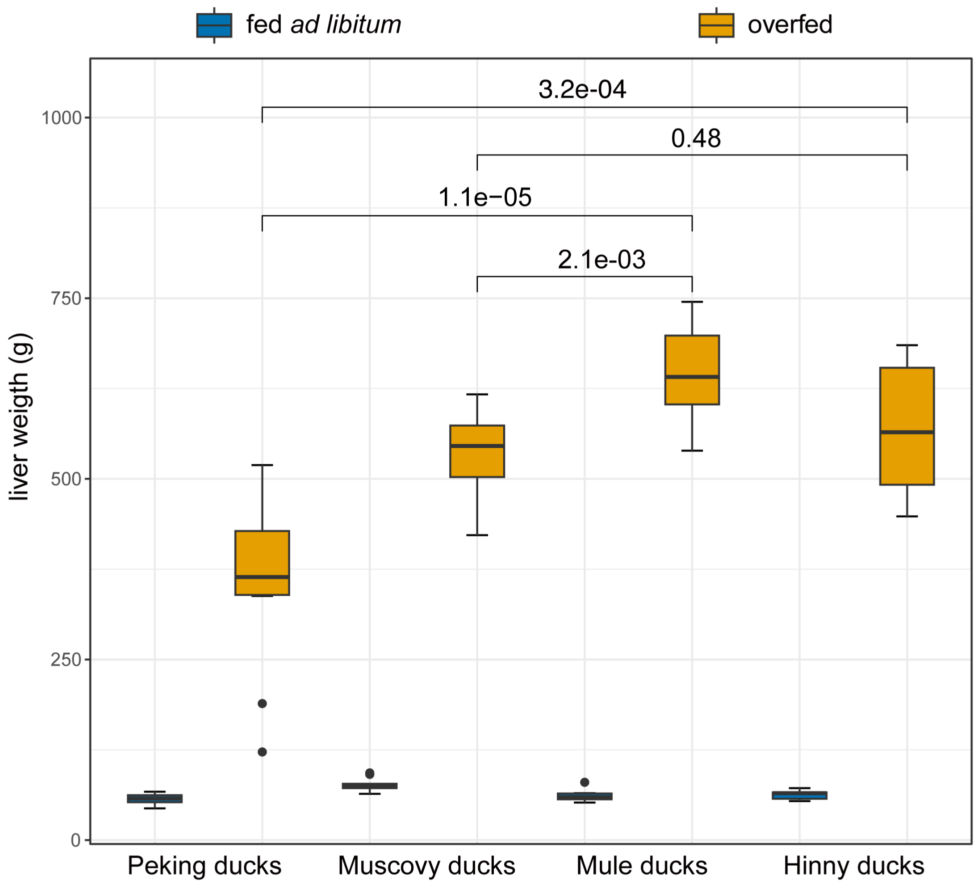

Supplementary Figure 7. Liver weights of four genotypes of ducks before and after overfeeding. Liver weights are shown for Peking, Muscovy, Mule, and Hinny ducks under *ad libitum* and overfed conditions. P-values were calculated using Wilcoxon test and are shown at the top of each comparison. The error bars indicate the whiskers of the boxplot, defined as the most extreme data points within 1.5 × the IQR from the Q1 and Q3 quartiles.

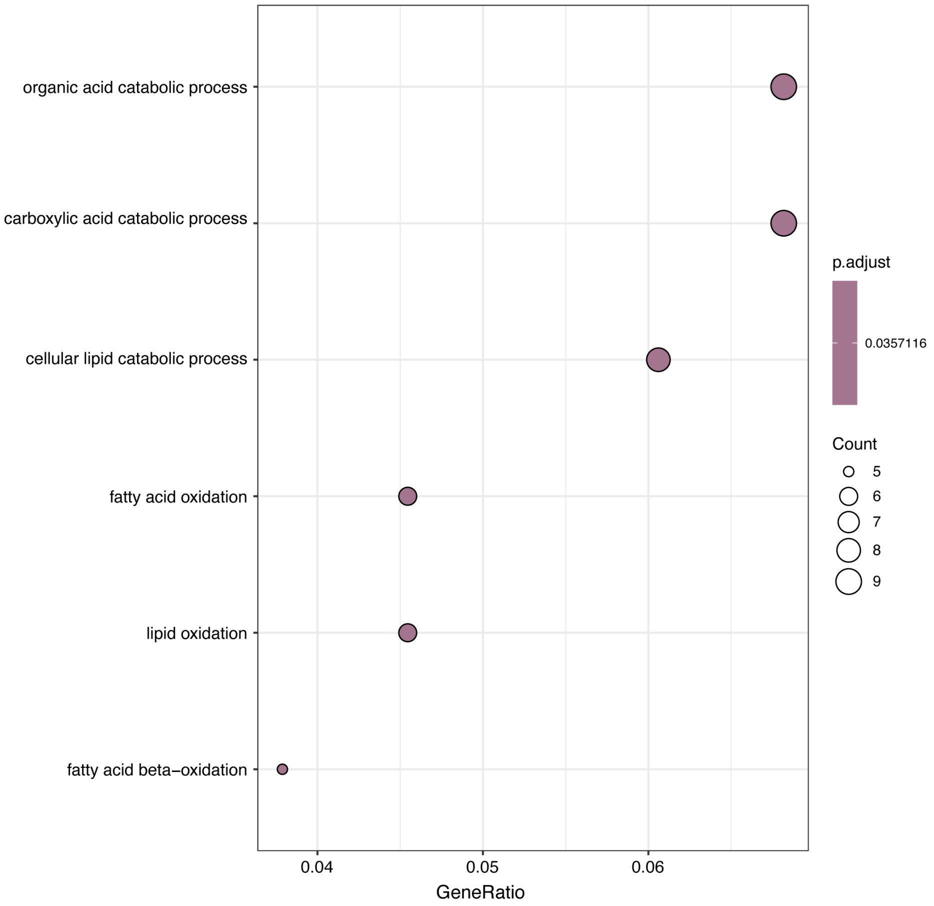

Supplementary Figure 8. Enriched GO terms associated with lipid catabolism were identified in Peking-to-Muscovy (PPAs) in the livers of Hinny ducks.

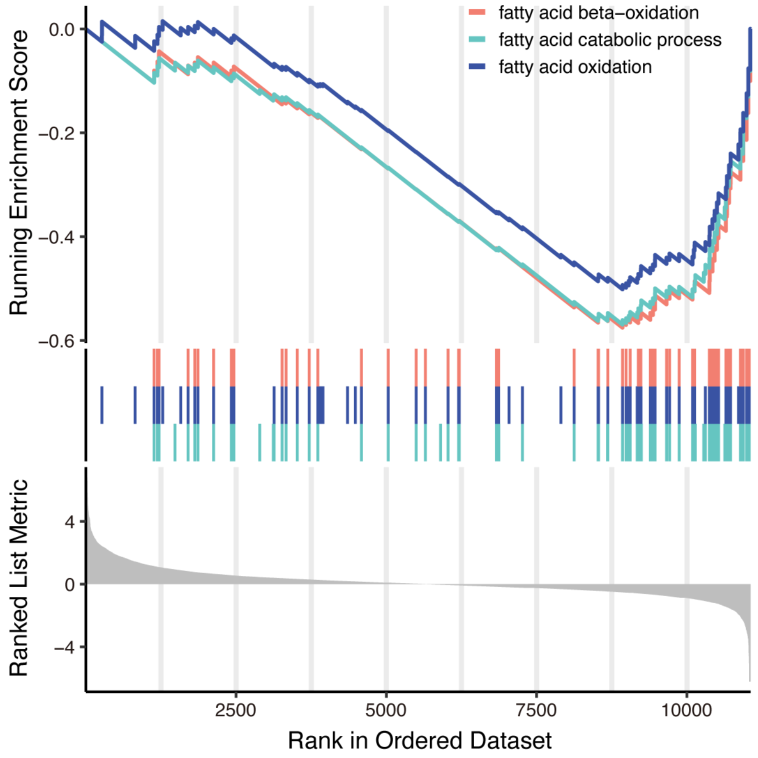

Supplementary Figure 9. GSEA shows that the lipid catabolism pathway, as represented by alleles from Peking ducks, were downregulated in livers of Hinny ducks.

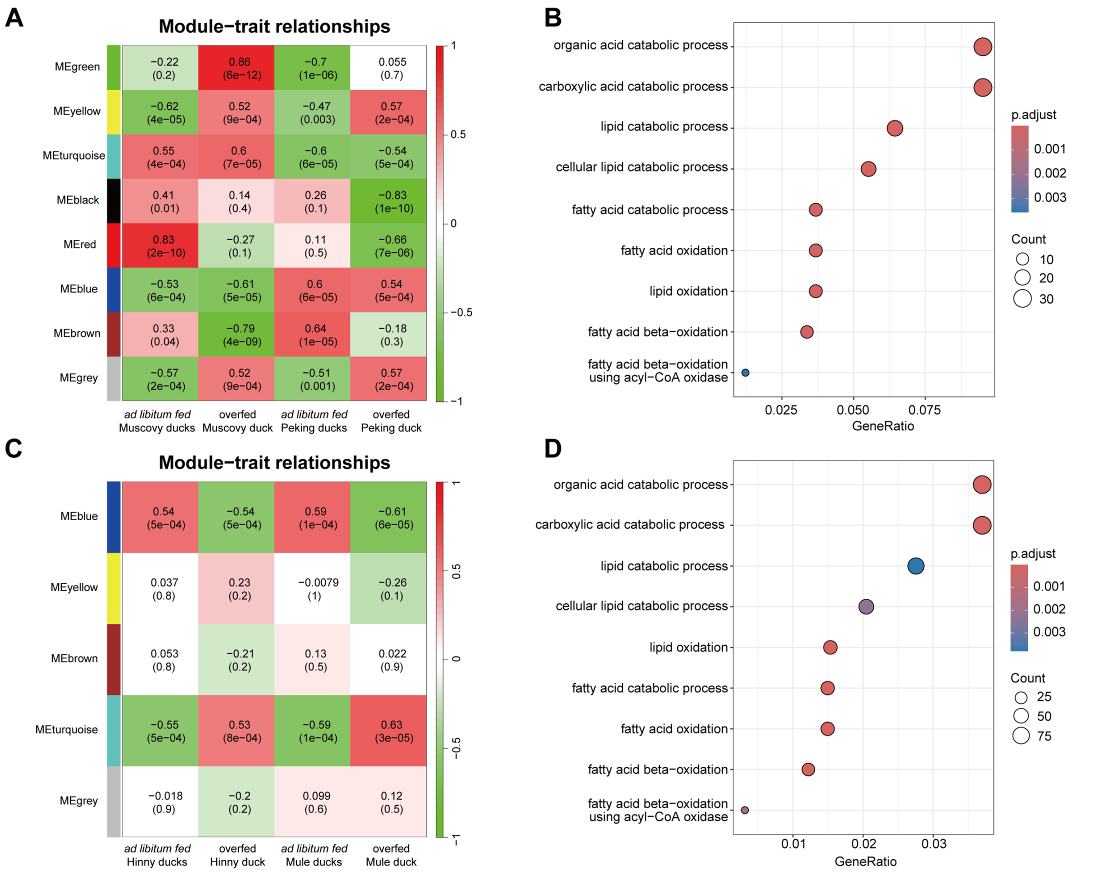

Supplementary Figure 10. Co-expression module–trait associations and functional enrichment analysis for parental and hybrid ducks under two feeding conditions. (**A, C**) Heatmap showing module-trait relationships identified by weighted gene co-expression network analysis (WGCNA) for parental ducks (**A**) and hybrid ducks (**C**). Each cell displays the correlation between a co-expression module eigengene and the indicated trait, with the correlation coefficient shown on the first line and the corresponding P value on the second line. Color bar reflects the strength and direction of the correlation (red, positive; green, negative). (**B, D**) Gene Ontology (GO) enrichment analysis of genes from the significant module for parental ducks (**B**) and hybrid ducks (**D**).

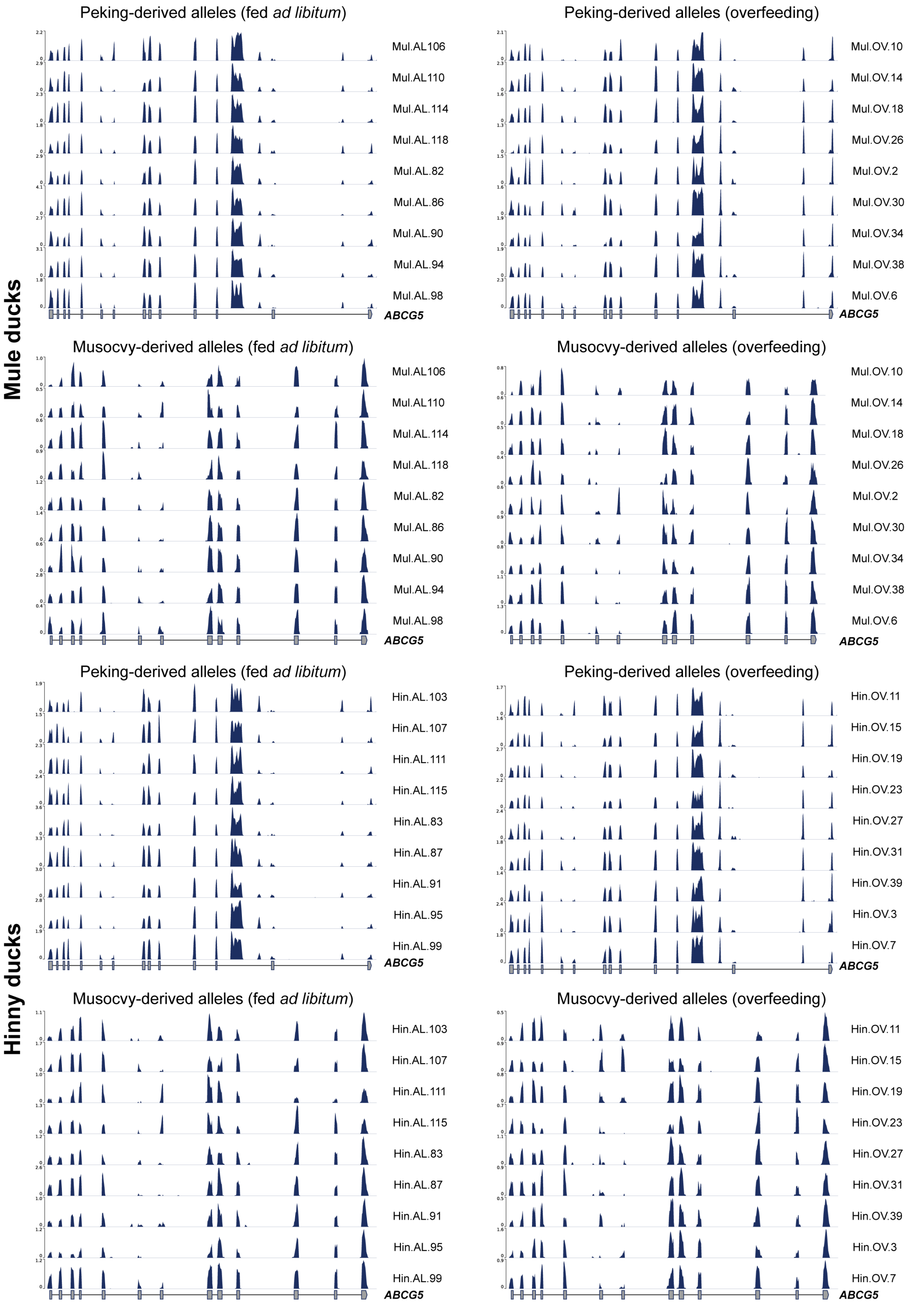
Mule ducks
Hinny ducks
Peking-derived alleles (fed *ad libitum*)
Peking-derived alleles (overfeeding)
Musocvy-derived alleles (fed *ad libitum*)
Musocvy-derived alleles (overfeeding)
Mul.AL106
Mul.AL110
Mul.AL.114
Mul.AL.118
Mul.AL.82
Mul.AL.86
Mul.AL.90
Mul.AL.94
Mul.AL.98
Mul.OV.10
Mul.OV.14
Mul.OV.18
Mul.OV.26
Mul.OV.2
Mul.OV.30
Mul.OV.34
Mul.OV.38
Mul.OV.6
Hin.AL.103
Hin.AL.107
Hin.AL.111
Hin.AL.115
Hin.AL.83
Hin.AL.87
Hin.AL.91
Hin.AL.95
Hin.AL.99
Hin.OV.11
Hin.OV.15
Hin.OV.19
Hin.OV.23
Hin.OV.27
Hin.OV.31
Hin.OV.39
Hin.OV.3
Hin.OV.7
ABCG5

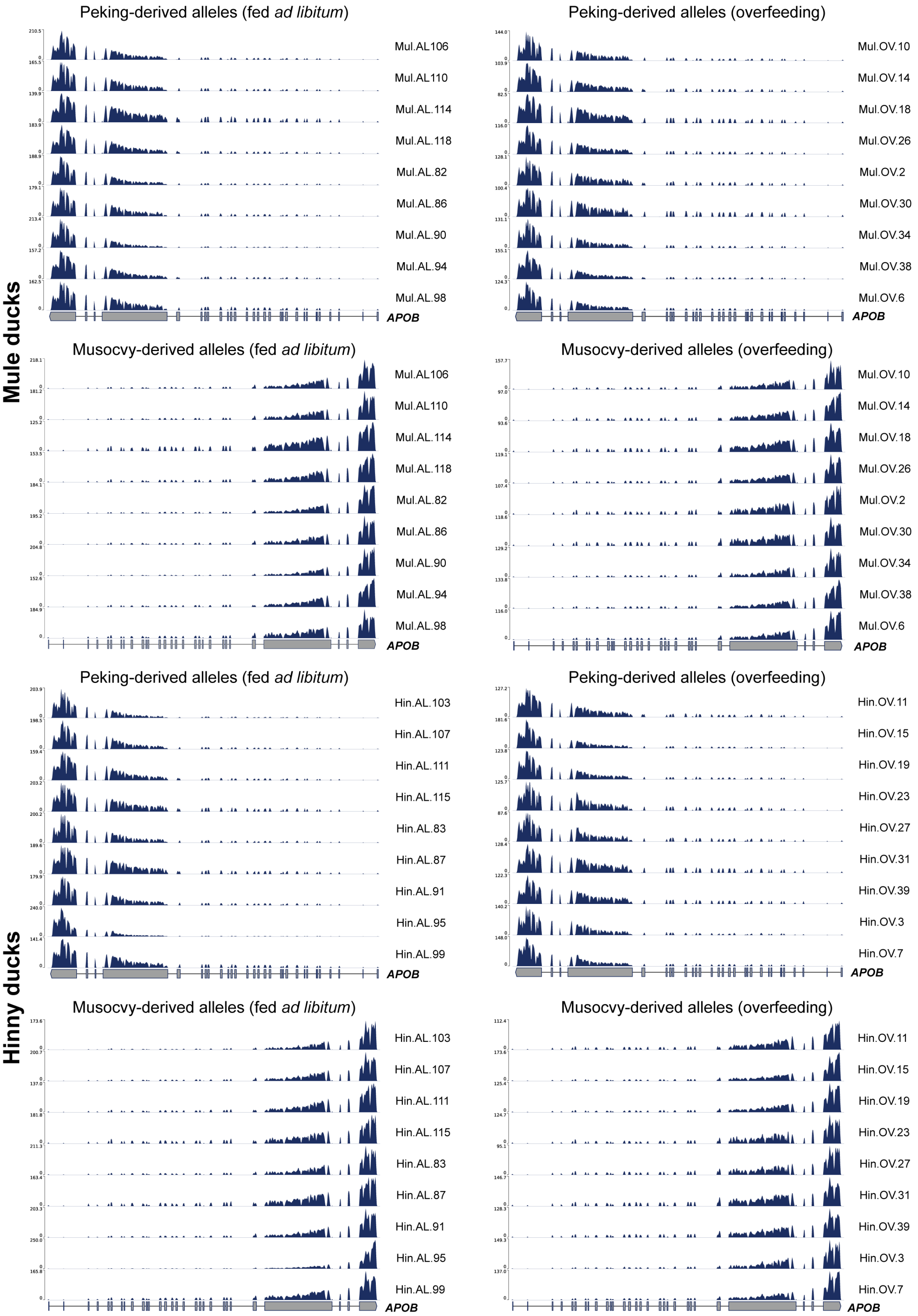
Mule ducks
Peking-derived alleles (fed *ad libitum*)
Mul.AL106
Mul.AL110
Mul.AL.114
Mul.AL.118
Mul.AL.82
Mul.AL.86
Mul.AL.90
Mul.AL.94
Mul.AL.98
APOB
Peking-derived alleles (overfeeding)
Mul.OV.10
Mul.OV.14
Mul.OV.18
Mul.OV.26
Mul.OV.2
Mul.OV.30
Mul.OV.34
Mul.OV.38
Mul.OV.6
APOB
Musocvy-derived alleles (fed *ad libitum*)
Mul.AL106
Mul.AL110
Mul.AL.114
Mul.AL.118
Mul.AL.82
Mul.AL.86
Mul.AL.90
Mul.AL.94
Mul.AL.98
APOB
Musocvy-derived alleles (overfeeding)
Mul.OV.10
Mul.OV.14
Mul.OV.18
Mul.OV.26
Mul.OV.2
Mul.OV.30
Mul.OV.34
Mul.OV.38
Mul.OV.6
APOB
Hinny ducks
Peking-derived alleles (fed *ad libitum*)
Hin.AL.103
Hin.AL.107
Hin.AL.111
Hin.AL.115
Hin.AL.83
Hin.AL.87
Hin.AL.91
Hin.AL.95
Hin.AL.99
APOB
Peking-derived alleles (overfeeding)
Hin.OV.11
Hin.OV.15
Hin.OV.19
Hin.OV.23
Hin.OV.27
Hin.OV.31
Hin.OV.39
Hin.OV.3
Hin.OV.7
APOB
Musocvy-derived alleles (fed *ad libitum*)
Hin.AL.103
Hin.AL.107
Hin.AL.111
Hin.AL.115
Hin.AL.83
Hin.AL.87
Hin.AL.91
Hin.AL.95
Hin.AL.99
APOB
Musocvy-derived alleles (overfeeding)
Hin.OV.11
Hin.OV.15
Hin.OV.19
Hin.OV.23
Hin.OV.27
Hin.OV.31
Hin.OV.39
Hin.OV.3
Hin.OV.7
APOB

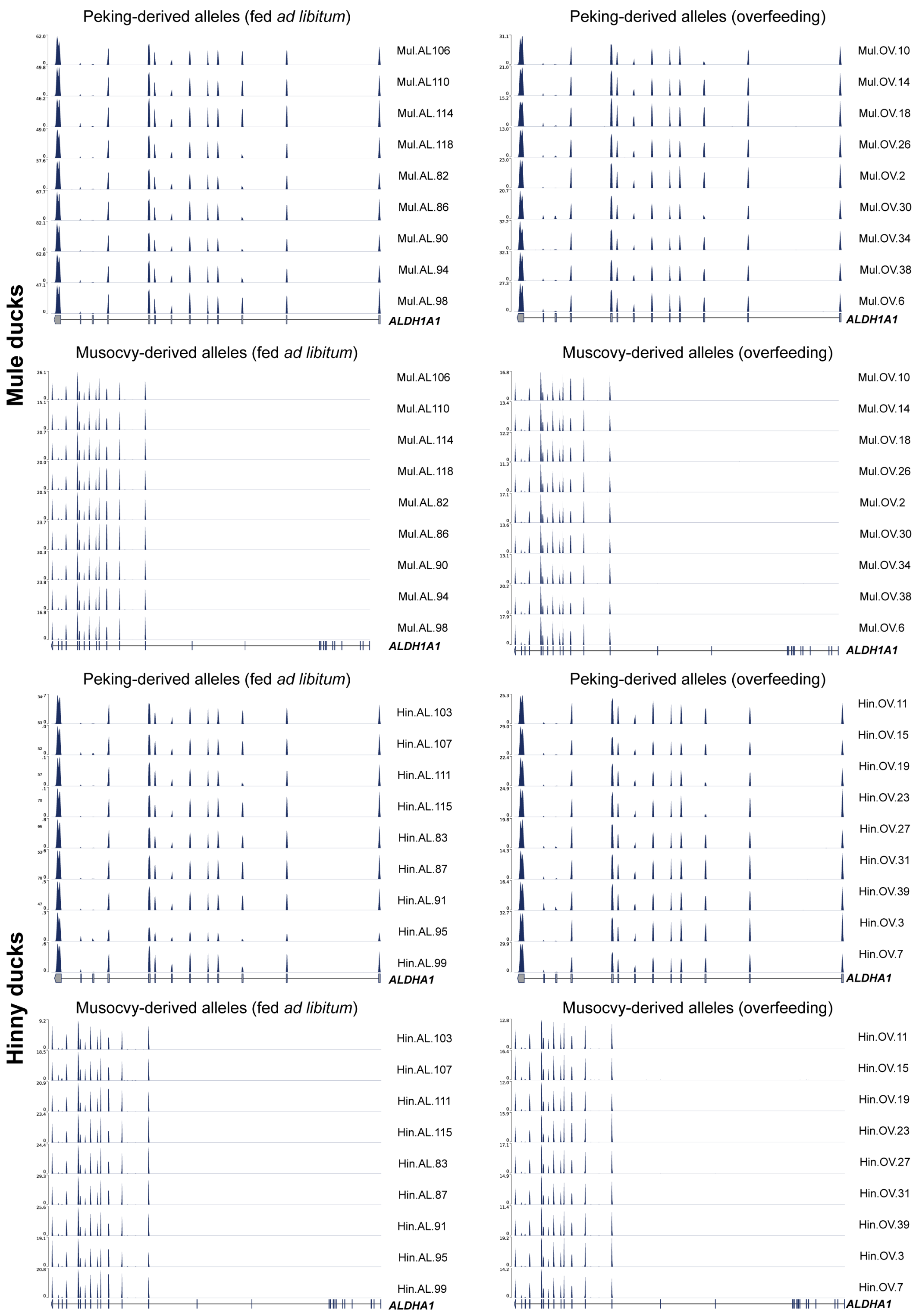
Mule ducks
Peking-derived alleles (fed ad libitum)
Mul.AL106
Mul.AL110
Mul.AL.114
Mul.AL.118
Mul.AL.82
Mul.AL.86
Mul.AL.90
Mul.AL.94
Mul.AL.98
ALDH1A1
Peking-derived alleles (overfeeding)
Mul.OV.10
Mul.OV.14
Mul.OV.18
Mul.OV.26
Mul.OV.2
Mul.OV.30
Mul.OV.34
Mul.OV.38
Mul.OV.6
ALDH1A1
Musocvy-derived alleles (fed ad libitum)
Mul.AL106
Mul.AL110
Mul.AL.114
Mul.AL.118
Mul.AL.82
Mul.AL.86
Mul.AL.90
Mul.AL.94
Mul.AL.98
ALDH1A1
Muscovy-derived alleles (overfeeding)
Mul.OV.10
Mul.OV.14
Mul.OV.18
Mul.OV.26
Mul.OV.2
Mul.OV.30
Mul.OV.34
Mul.OV.38
Mul.OV.6
ALDH1A1
Hinny ducks
Peking-derived alleles (fed ad libitum)
Hin.AL.103
Hin.AL.107
Hin.AL.111
Hin.AL.115
Hin.AL.83
Hin.AL.87
Hin.AL.91
Hin.AL.95
Hin.AL.99
ALDHA1
Peking-derived alleles (overfeeding)
Hin.OV.11
Hin.OV.15
Hin.OV.19
Hin.OV.23
Hin.OV.27
Hin.OV.31
Hin.OV.39
Hin.OV.3
Hin.OV.7
ALDHA1
Musocvy-derived alleles (fed ad libitum)
Hin.AL.103
Hin.AL.107
Hin.AL.111
Hin.AL.115
Hin.AL.83
Hin.AL.87
Hin.AL.91
Hin.AL.95
Hin.AL.99
ALDHA1
Musocvy-derived alleles (overfeeding)
Hin.OV.11
Hin.OV.15
Hin.OV.19
Hin.OV.23
Hin.OV.27
Hin.OV.31
Hin.OV.39
Hin.OV.3
Hin.OV.7
ALDHA1

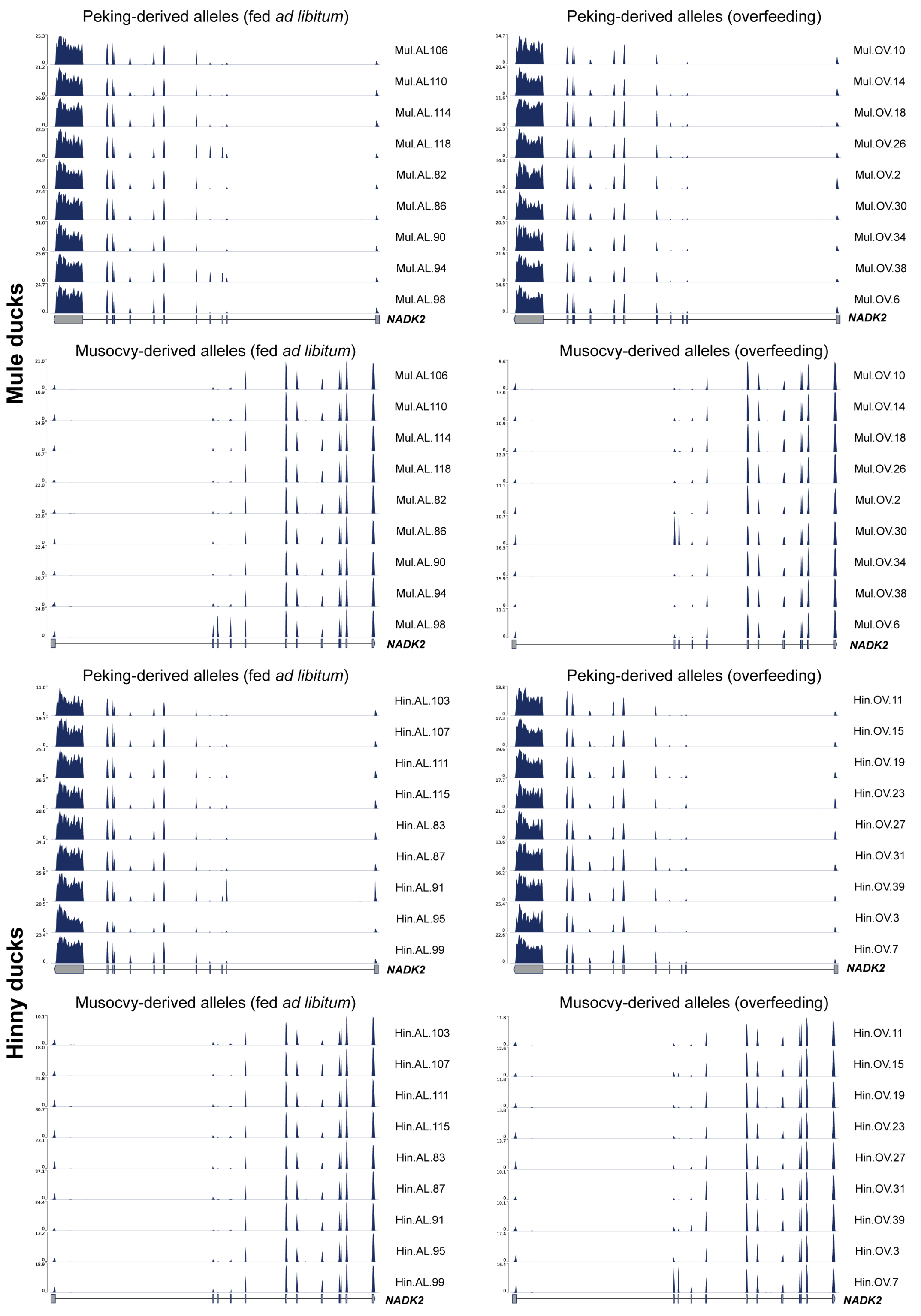

Supplementary Figure 11. Distribution of RNA-seq read coverage across the exonic regions of candidate genes.

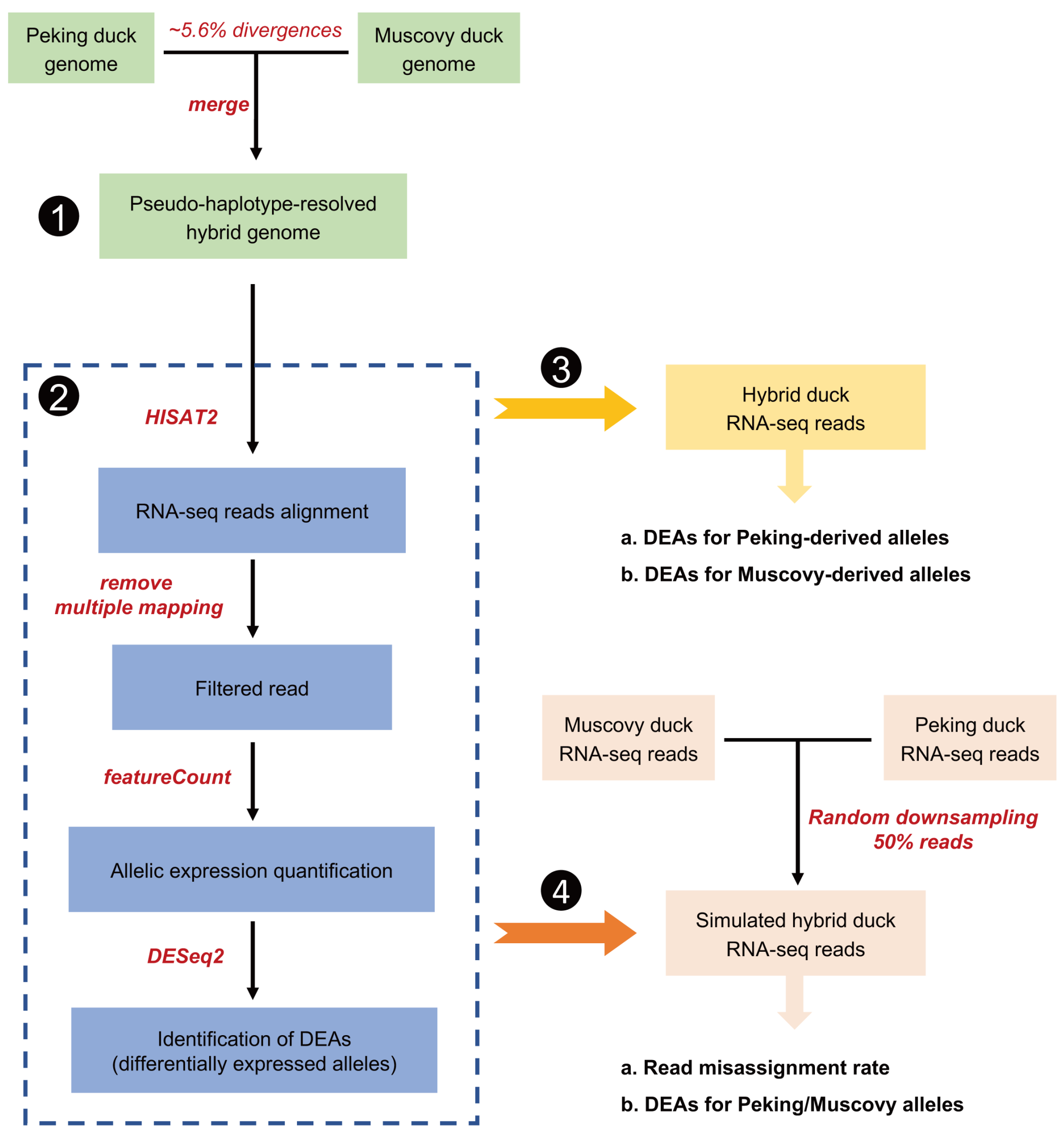

Supplementary Figure 12. Workflow for the analysis of hepatic transcriptomic data in hybrids.

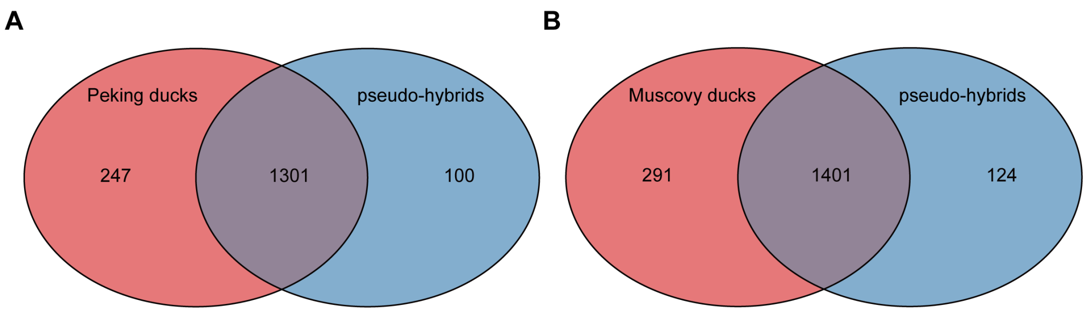

Supplementary Figure 13. Consistent plastic changes in gene expression between parental duck species and simulated pseudo-hybrids in response to overfeeding. (**A**) Venn plot showing the intersection between differentially expressed genes (DEGs) of Peking ducks and Peking-derived alleles in simulated pseudo-hybrids. (**B**) Venn plot showing the intersection between DEGs of Muscovy ducks and Muscovy-derived alleles in simulated pseudo-hybrids.

Supplementary Tables

Supplementary Table 1. The differentially expressed genes between parental and hybrid ducks

	fed *ad libitum*		overfeeding	
	Peling ducks	Muscovy ducks	Peling ducks	Muscovy ducks
Mule ducks	575	1,406	602	1,834
Hinny ducks	576	1,346	630	1,780

Supplementary Table 2. The percentage of heterosis for Mule and Hinny ducks

	fed *ad libitum*		overfeeding	
	Mule ducks	Hinny ducks	Mule ducks	Hinny ducks
H%	-8.14	-5.6	45.72	28.04
t-statistic	-45.68	-39.7	75.31	33.66
p-value	1	1	3.24E-14	4.45E-11

Supplementary Table 3. Summary statistics of cross-species RNA-seq read alignment between Peking and Muscovy ducks

Sample	Total reads	Alignment to Peking duck genome		Alignment to Muscovy duck genome	
		Properly aligned reads	Properly aligned rate	Properly aligned reads	Properly aligned rate
Pek.AL.101	29,773,930	25,911,470	87.03%	18,759,660	63.01%
Pek.AL.105	65,912,956	59,047,332	89.58%	42,713,194	64.80%
Pek.AL.109	64,942,634	56,795,282	87.45%	38,792,460	59.73%
Pek.AL.113	41,516,114	36,347,414	87.55%	25,097,002	60.45%
Pek.AL.117	40,973,316	36,035,776	87.95%	24,981,100	60.97%
Pek.AL.81	54,511,592	49,032,468	89.95%	35,977,162	66.00%
Pek.AL.85	63,672,484	57,586,492	90.44%	42,128,456	66.16%
Pek.AL.89	54,632,570	48,994,736	89.68%	35,488,578	64.96%
Pek.AL.93	100,722,690	90,183,994	89.54%	65,498,056	65.03%
Pek.AL.97	62,509,638	56,142,884	89.81%	40,555,724	64.88%
Pek.OV.13	73,132,064	65,232,488	89.20%	45,329,446	61.98%
Pek.OV.17	42,987,014	37,949,470	88.28%	24,365,680	56.68%
Pek.OV.1	49,425,436	43,075,334	87.15%	29,901,862	60.50%
Pek.OV.21	39,875,958	35,196,206	88.26%	23,784,718	59.65%
Pek.OV.25	40,240,106	35,000,736	86.98%	22,779,380	56.61%
Pek.OV.29	60,005,478	52,610,462	87.68%	35,564,472	59.27%
Pek.OV.33	62,297,874	54,791,324	87.95%	35,097,266	56.34%
Pek.OV.37	40,498,382	34,885,134	86.14%	22,813,904	56.33%
Pek.OV.5	63,881,908	55,665,810	87.14%	36,431,272	57.03%
Pek.OV.9	58,273,928	50,654,926	86.93%	36,093,026	61.94%
Mus.AL.104	52,982,008	32,626,890	61.58%	45,380,938	85.65%
Mus.AL.108	43,990,130	25,078,018	57.01%	36,374,144	82.69%
Mus.AL.112	46,767,682	26,598,310	56.87%	38,809,774	82.98%
Mus.AL.116	53,249,208	32,086,080	60.26%	44,995,450	84.50%
Mus.AL.120	47,410,634	28,176,254	59.43%	39,726,656	83.79%
Mus.AL.84	57,399,836	34,621,410	60.32%	48,685,078	84.82%
Mus.AL.88	42,570,376	25,968,518	61.00%	36,177,778	84.98%
Mus.AL.92	44,882,126	26,407,674	58.84%	37,488,010	83.53%
Mus.AL.96	59,719,512	37,480,686	62.76%	51,126,034	85.61%
Mus.OV.12	58,607,268	32,595,598	55.62%	47,463,426	80.99%
Mus.OV.16	62,651,190	34,146,672	54.50%	50,885,728	81.22%
Mus.OV.20	61,531,550	35,558,148	57.79%	50,613,346	82.26%
Mus.OV.24	52,439,560	31,915,772	60.86%	44,295,778	84.47%
Mus.OV.28	40,255,992	21,672,382	53.84%	32,279,306	80.19%
Mus.OV.32	45,563,618	27,778,676	60.97%	38,453,580	84.40%
Mus.OV.36	53,283,106	31,979,646	60.02%	44,537,482	83.59%
Mus.OV.40	54,695,162	32,225,370	58.92%	46,149,840	84.38%
Mus.OV.4	59,203,318	35,180,604	59.42%	49,133,516	82.99%

Mus.OV.8	90,014,240	51,720,612	57.46%	74,450,752	82.71%
