# Supplementary material for: Restoration of ancestral transcriptional plasticity contributes to plastic heterosis in fatty liver of hybrid ducks
Source: Commun Biol. 2026 Apr 14;9:803. doi: 10.1038/s42003-026-10049-7 (PMC13266055; doi:10.1038/s42003-026-10049-7)
Supplement: Supplementary file 2 — Description of Additional Supplementary Files [file 42003_2026_10049_MOESM2_ESM.pdf]

Description of Additional Supplementary Files

File name: Supplementary Information

Description: The PDF of all supplementary Figure

File name: Supplementary data 1

Description: GO enrichment results for plastic genes shared by Peking and Muscovy ducks

File name: Supplementary data 2

Description: GO enrichment results for downregulated and upregulated plastic genes specific to Muscovy and Peking ducks

File name: Supplementary data 3

Description: GO enrichment results for downregulated and upregulated ancestral plastic genes

File name: Supplementary data 4

Description: GO enrichment results for intersected Peking-to-Muscovy PPAs between Mule and Hinny ducks

File name: Supplementary data 5

Description: Summary of samples and sequencing data used in this study

File name: Supplementary data 6

Description: Summary of reference genome information used in this study

File name: Supplementary data 7

Description: Summary of orthologous gene sets among Peking ducks, Muscovy ducks, and geese

File name: Supplementary data 8

Description: Read assignment statistics for simulated pseudo-hybrid RNA-seq datasets
