# Supplementary material for: Restoration of ancestral transcriptional plasticity contributes to plastic heterosis in fatty liver of hybrid ducks
Source: Commun Biol. 2026 Apr 14;9:803. doi: 10.1038/s42003-026-10049-7 (PMC13266055; doi:10.1038/s42003-026-10049-7)
Supplement: Supplementary file 11 — Reporting_summary [file 42003_2026_10049_MOESM11_ESM.pdf]

nature portfolio

Corresponding author(s): DAPR COMMSBIO-25-6986A

Last updated by author(s): Mar 3rd, 2026

Reporting Summary

Nature Portfolio wishes to improve the reproducibility of the work that we publish. This form provides structure for consistency and transparency in reporting. For further information on Nature Portfolio policies, see our Editorial Policies and the Editorial Policy Checklist.

Statistics

For all statistical analyses, confirm that the following items are present in the figure legend, table legend, main text, or Methods section.

n/a	Confirmed	
☐	☒	The exact sample size (*n*) for each experimental group/condition, given as a discrete number and unit of measurement
☐	☒	A statement on whether measurements were taken from distinct samples or whether the same sample was measured repeatedly
☐	☒	The statistical test(s) used AND whether they are one- or two-sided<br>*Only common tests should be described solely by name; describe more complex techniques in the Methods section.*
☐	☒	A description of all covariates tested
☐	☒	A description of any assumptions or corrections, such as tests of normality and adjustment for multiple comparisons
☒	☐	A full description of the statistical parameters including central tendency (e.g. means) or other basic estimates (e.g. regression coefficient) AND variation (e.g. standard deviation) or associated estimates of uncertainty (e.g. confidence intervals)
☐	☒	For null hypothesis testing, the test statistic (e.g. *F*, *t*, *r*) with confidence intervals, effect sizes, degrees of freedom and *P* value noted<br>*Give P values as exact values whenever suitable.*
☒	☐	For Bayesian analysis, information on the choice of priors and Markov chain Monte Carlo settings
☒	☐	For hierarchical and complex designs, identification of the appropriate level for tests and full reporting of outcomes
☒	☐	Estimates of effect sizes (e.g. Cohen's *d*, Pearson's *r*), indicating how they were calculated

Our web collection on statistics for biologists contains articles on many of the points above.

Software and code

Policy information about availability of computer code

Data collection	Codes used to analyze results in this manuscript are available on GitHub at https://github.com/qiuyixmm/Plastic-Heterosis-Paper.
Data analysis	Codes used to analyze results in this manuscript are available on GitHub at https://github.com/qiuyixmm/Plastic-Heterosis-Paper.

For manuscripts utilizing custom algorithms or software that are central to the research but not yet described in published literature, software must be made available to editors and reviewers. We strongly encourage code deposition in a community repository (e.g. GitHub). See the Nature Portfolio guidelines for submitting code & software for further information.

Data

Policy information about availability of data

All manuscripts must include a data availability statement. This statement should provide the following information, where applicable:

- Accession codes, unique identifiers, or web links for publicly available datasets
- A description of any restrictions on data availability
- For clinical datasets or third party data, please ensure that the statement adheres to our policy

All data supporting the findings of this study are publicly available. The transcriptomic data of Peking, Muscovy, Mule and Hinny ducks are available in the NCBI Sequence Read Archive under accession number SRP144764. The transcriptomic data of geese are available in the Gene Expression Omnibus under accession number GSE119421.

Research involving human participants, their data, or biological material

Policy information about studies with human participants or human data. See also policy information about sex, gender (identity/presentation), and sexual orientation and race, ethnicity and racism.

Reporting on sex and gender	N/A
Reporting on race, ethnicity, or other socially relevant groupings	N/A
Population characteristics	N/A
Recruitment	N/A
Ethics oversight	N/A

Note that full information on the approval of the study protocol must also be provided in the manuscript.

Field-specific reporting

Please select the one below that is the best fit for your research. If you are not sure, read the appropriate sections before making your selection.

☐ Life sciences ☐ Behavioural & social sciences ☒ Ecological, evolutionary & environmental sciences

For a reference copy of the document with all sections, see nature.com/documents/nr-reporting-summary-flat.pdf

Ecological, evolutionary & environmental sciences study design

All studies must disclose on these points even when the disclosure is negative.

Study description	The development of fatty liver in Muscovy and Peking ducks after overfeeding represents a typical case of phenotypic plasticity. The heterosis of fatty liver observed in their reciprocal interspecific hybrids (Mule and Hinny ducks) is a representative example of plastic heterosis. To investigate the evolutionary trajectory of phenotypic plasticity, we first performed comparative transcriptomic analyses of the parental species. We then characterized the transcriptional profiles of the hybrid ducks to examine how parental plasticity is reshaped through hybridization. Finally, we evaluated the impact of parental plasticity on plastic heterosis in hybrids and propose a novel mechanism underlying this phenomenon.
Research sample	The research samples consisted of Peking ducks (Anas platyrhynchos), Muscovy ducks (Cairina moschata), and their reciprocal interspecific hybrids (Mule and Hinny ducks), as well as geese (Anser cygnoides). Transcriptomic data were derived from liver tissues under ad libitum feeding and overfeeding conditions. All of these data were obtained from publicly available datasets.
Sampling strategy	To ensure consistent replication and maximize the robustness of transcriptomic analyses, nine biological replicates were selected for both the parental duck species and the hybrid ducks. For geese, transcriptomic data were obtained from a single study with three replicates in order to avoid unnecessary batch effects.
Data collection	No additional data collection procedures were required, as all transcriptomic data were obtained from publicly available datasets.
Timing and spatial scale	As all transcriptomic data were obtained from publicly available repositories, no new sample collection was performed in this study. The timing of data collection corresponds to the original studies that generated these datasets. Spatial scale is not applicable, as the data were derived from controlled laboratory experiments rather than field sampling.
Data exclusions	No data were excluded from the analysis.
Reproducibility	All analyses were performed using publicly available transcriptomic datasets with clearly defined accession numbers. Replicates were consistently included for each parental and hybrid group (nine biological replicates for ducks; three biological replicates for geese). Standardized bioinformatic pipelines were applied, and all results can be reproduced using the same datasets and described methods.
Randomization	Randomization is not applicable. The transcriptomic data for ducks and geese were divided into ad libitum fed and overfed groups in order to characterize transcriptional plasticity between the two feeding conditions.
Blinding	No blinding was performed, as all transcriptomic data were obtained from publicly available repositories. Data processing and analysis were conducted using standardized bioinformatic pipelines, which do not involve subjective assessment.

Did the study involve field work? ☐ Yes ☒ No

Reporting for specific materials, systems and methods

We require information from authors about some types of materials, experimental systems and methods used in many studies. Here, indicate whether each material, system or method listed is relevant to your study. If you are not sure if a list item applies to your research, read the appropriate section before selecting a response.

Materials & experimental systems

n/a	Involved in the study
☒	☐ Antibodies
☒	☐ Eukaryotic cell lines
☒	☐ Palaeontology and archaeology
☒	☐ Animals and other organisms
☒	☐ Clinical data
☒	☐ Dual use research of concern
☒	☐ Plants

Methods

n/a	Involved in the study
☒	☐ ChIP-seq
☒	☐ Flow cytometry
☒	☐ MRI-based neuroimaging

Plants

Seed stocks	This does not apply.
Novel plant genotypes	This does not apply.
Authentication	This does not apply.
